# SWI/SNF regulates a transcriptional program that induces senescence to prevent liver cancer

**DOI:** 10.1101/gad.286112.116

**Published:** 2016-10-01

**Authors:** Luca Tordella, Sadaf Khan, Anja Hohmeyer, Ana Banito, Sabrina Klotz, Selina Raguz, Nadine Martin, Gopuraja Dhamarlingam, Thomas Carroll, José Mario González Meljem, Sumit Deswal, Juan Pedro Martínez-Barbera, Ramón García-Escudero, Johannes Zuber, Lars Zender, Jesús Gil

**Affiliations:** 1 MRC Clinical Sciences Centre (CSC), London W12 0NN, United Kingdom;; 2 Institute of Clinical Sciences (ICS), Faculty of Medicine Imperial College London, London W12 0NN, United Kingdom;; 3 Division of Molecular Oncology of Solid Tumours, Department of Internal Medicine I, Eberhard Karls University Tübingen, 72076 Tübingen, Germany;; 4 Developmental Biology and Cancer Programme, Birth Defects Research Centre, University College London Institute of Child Health, London WC1N 1EH, United Kingdom;; 5 Research Institute of Molecular Pathology (IMP), 1030 Vienna, Austria;; 6 Molecular Oncology Unit, Centro de Investigaciones Energéticas, Medioambientales y Tecnológicas (CIEMAT), 28040 Madrid, Spain;; 7 Biomedical Research Institute I+12, University Hospital 12 de Octubre, 28041 Madrid, Spain

**Keywords:** senescence, ARID1B, SWI/SNF, p53, ENTPD7, dNTP metabolism, cancer

## Abstract

Oncogene-induced senescence (OIS) is a potent tumor suppressor mechanism. To identify senescence regulators relevant to cancer, we screened an shRNA library targeting genes deleted in hepatocellular carcinoma (HCC). Here, we describe how knockdown of the SWI/SNF component ARID1B prevents OIS and cooperates with RAS to induce liver tumors. ARID1B controls p16^INK4a^ and p21^CIP1a^ transcription but also regulates DNA damage, oxidative stress, and p53 induction, suggesting that SWI/SNF uses additional mechanisms to regulate senescence. To systematically identify SWI/SNF targets regulating senescence, we carried out a focused shRNA screen. We discovered several new senescence regulators, including ENTPD7, an enzyme that hydrolyses nucleotides. ENTPD7 affects oxidative stress, DNA damage, and senescence. Importantly, expression of ENTPD7 or inhibition of nucleotide synthesis in ARID1B-depleted cells results in re-establishment of senescence. Our results identify novel mechanisms by which epigenetic regulators can affect tumor progression and suggest that prosenescence therapies could be employed against SWI/SNF-mutated cancers.

## Abstract (precis)

Here, Tordella et. al identified senescence regulators relevant to cancer by screening an shRNA library targeting genes deleted in hepatocellular carcinoma (HCC). They show that knockdown of the SWI/SNF component ARID1B prevents oncogene-induced senescence and cooperates with RAS to induce liver tumors, and their results provide new insights into the mechanisms by which epigenetic regulators can affect tumor progression.

The extensive sequencing of cancer genomes has provided a comprehensive list of the recurrent genetic alterations observed in different tumor types. In addition to well-known oncogenes and tumor suppressors, these studies have linked unforeseen candidates to cancer (Watson et al. 2013). In parallel, our overall understanding of cancer biology has advanced considerably, and specific hallmarks have been proposed to explain the diversity of cancer (Hanahan and Weinberg 2011). However, despite progress in both areas, the explanation for the functional significance that recurring genetic alterations have in cancer progression is often missing but is much needed to develop targeted therapies.

Bypass of senescence is a common characteristic of advanced tumors (Narita and Lowe 2005). In response to oncogenic signaling, cells activate a stress response termed oncogene-induced senescence (OIS). OIS is a feature of premalignant lesions and has an important role in tumor suppression (Collado and Serrano 2010). Consequently, advanced tumors almost invariably escape senescence, and this constitutes one of the hallmarks of cancer (Hanahan and Weinberg 2011). A well-studied example is hepatocellular carcinoma (HCC). Aberrant oncogenic activation in hepatocytes activates OIS, which in turn limits HCC generation via cell-intrinsic and cell-extrinsic mechanisms (Kang et al. 2011). Overall, OIS is a complex program that includes the implementation of a highly stable cell cycle arrest but also profound changes in transcription, metabolism, secretome, and chromatin organization (Kuilman et al. 2010). Many of these senescent phenotypes are under epigenetic control (Kia et al. 2008; Barradas et al. 2009). Interestingly, a major discovery from cancer genome studies is that chromatin modifiers are often mutated in cancer (Plass et al. 2013). In particular, genes encoding for components of the SWI/SNF chromatin remodeling complex are frequently deleted or mutated across a wide spectrum of cancers (Kadoch et al. 2013). This suggests that SWI/SNF plays a fundamental protective role toward tumorigenesis via the regulation of basic cellular functions. Whether the frequency of these alterations relates to the ability of the SWI/SNF complex to control senescence is an enticing possibility.

The SWI/SNF complexes reposition nucleosomes and bind to DNA regulatory regions to control transcription. Loss-of-function mutations of different SWI/SNF components drive tumorigenesis by disrupting gene expression (Wilson and Roberts 2011). SWI/SNF are macromolecular complexes comprising 12–15 subunits: A catalytic ATPase subunit, SMARCA4/BRG1 or SMARCA2/BRM, and several core subunits, such as SMARCB1/SNF5/INI1/BAF47 or SMARCC1/BAF155, are common to all SWI/SNF complexes. Other subunits, such as ARID1A and ARID1B, are mutually exclusive components of BAF (BRG1-associated factor) complexes, while PBRM1 and ARID2 are specific for PBAF (polybromo BAF) complexes (Helming et al. 2014a).

Many components of SWI/SNF complexes are altered in different malignancies, adding up to a global ∼20% of cancers bearing mutations in SWI/SNF genes (Kadoch et al. 2013). The BAF subunit ARID1A has the highest mutation rate among SWI/SNF components (Helming et al. 2014a). Mutations in its paralog, ARID1B, are less frequent but have also been observed in melanoma, gastric cancer, colorectal cancer, HCC, neuroblastoma, and pancreatic cancer (Fujimoto et al. 2012; Khursheed et al. 2013; Sausen et al. 2013; Lee et al. 2015). In HCC, >20% of the tumors present alterations in SWI/SNF components, and mutations in ARID1B are second only to ARID1A in frequency (Fujimoto et al. 2012).

Given the prevalence of SWI/SNF mutations, several studies have investigated the molecular mechanisms linking SWI/SNF disruption with cancer progression. SWI/SNF components have been reported to interact with RB (Trouche et al. 1997), MYC (Cheng et al. 1999), or p53 (Lee et al. 2002). Moreover, inactivation of the SWI/SNF complex due to loss of specific components such as SNF5, BRD7, or PBR1 results in senescence bypass (Oruetxebarria et al. 2004; Burrows et al. 2010; Drost et al. 2010). Direct transcriptional regulation of p16^INK4a^ (Oruetxebarria et al. 2004; Kia et al. 2008) or p21^CIP1^ (Chai et al. 2005) by SWI/SNF complexes might explain, in part, their role in senescence. However, a more comprehensive explanation of how SWI/SNF regulates senescence and how this is relevant for tumorigenesis is still lacking.

In this investigation, we carried out a functional screen to identify HCC-deleted genes regulating senescence. We found that depletion of ARID1B blunted the senescence response and cooperated with oncogenic Ras in HCC formation. By systematically investigating ARID1B target genes, we discovered several novel mediators of senescence, among them ENTPD7, an enzyme involved in nucleotide metabolism. Overall, the identification of novel mechanisms by which ARID1B loss restricts senescence could reveal vulnerabilities (e.g., nucleotide metabolism) useful to target cancers bearing mutations in SWI/SNF genes.

## Results

### An shRNA screen identifies Arid1b as a regulator of senescence

**Figure 1.**
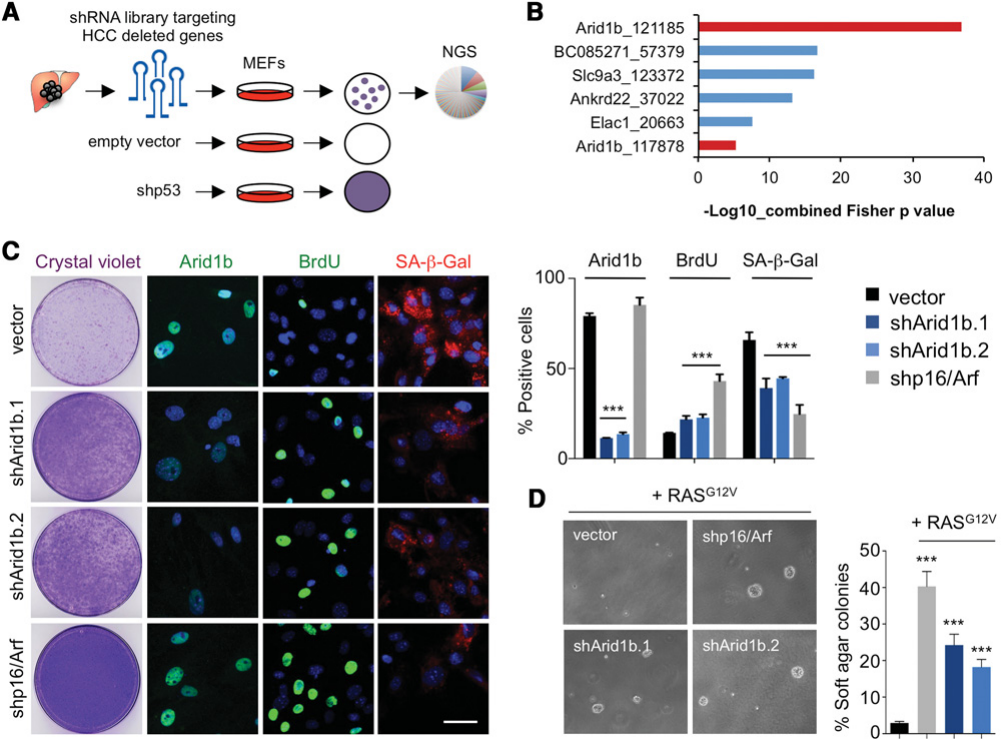
An shRNA screen identifies the SWI/SNF subunit ARID1B as a regulator of senescence. (*A*) Schematic model of the bypass of senescence screen using a library of shRNAs targeting genes deleted in HCC. An average of two different shRNAs per gene were used. MEFs were infected with the shRNA library or controls and passaged until vector cells reached senescence. shRNAs that extended life span were recovered by NGS. The screen was performed in triplicate. (*B*) List of significantly enriched shRNAs by Fisher's combined *P*-value (*P* < 0.001). shRNAs targeting ARID1B are marked in red. (*C*) Representative images of ARID1B, BrdU, SA-β-Gal immunofluorescence (IF) staining, and colony formation assay (crystal violet) of MEFs transduced with shRNAs targeting ARID1B, p16^INK4a^, or empty vector. DAPI was used to visualize the nuclei. Bar, 30 μm. Quantification of IF by high-content analysis (HCA) is shown at the *right*. (*D*) Soft agar assay of MEFs transduced with *Ras*^*G12V*^ cDNA and either an empty vector or vectors carrying shRNAs for p16/Arf or ARID1B. Representative images at 4× magnification (*left*) and quantification (*right*) are shown. Graphs in *C* and *D* represent mean ± SD from *n* = 4 and *n* = 3, respectively. (***) *P* < 0.001 by two-tailed Student *t*-test.

Senescence is a potent tumor suppressor mechanism (Kuilman et al. 2010) often disabled in cancer cells (Hanahan and Weinberg 2011). For example, induction of senescence in preneoplastic hepatocytes actively prevents HCC (Kang et al. 2011). To identify tumor suppressors that control senescence and could limit liver cancer, we devised a genetic screen using an shRNA library targeting genes frequently deleted in HCC (Zender et al. 2008). We carried out the screen in mouse embryonic fibroblasts (MEFs), which are a robust system used previously to identify regulators of senescence (Jacobs et al. 2000; Kondoh et al. 2005). MEFs were infected with the shRNA library, selected, and passaged regularly until controls underwent senescence (Fig. 1A; Supplemental Fig. S1A). The relative distribution of shRNAs during the screen was determined using next-generation sequencing (NGS). We then used statistical analysis to identify shRNAs significantly enriched in MEFs bypassing senescence (Fig. 1B; Supplemental Fig. S1B). Among the enriched shRNAs, two targeted the SWI/SNF subunit *Arid1b* (including the top shRNA enriched in the screen) (Fig. 1B; Supplemental Fig. S1B). Since SWI/SNF components are frequently mutated or deleted in cancer (Oruetxebarria et al. 2004; Shain and Pollack 2013), we decided to confirm that Arid1b knockdown prevented senescence using two shRNAs targeting murine *Arid1b* (Fig. 1C; Supplemental Fig. S1C,D). Similar to what we observed when using an shRNA targeting *Ink4a/Arf* (shp16/Arf) (Supplemental Fig. S1C), knockdown of *Arid1b* blunted replicative senescence, as evidenced by increased colony formation, a higher percentage of cells incorporating BrdU, and a decrease in the percentage of senescence-associated β-galactosidase (SA-β-Gal)-positive cells when compared with control MEFs (Fig. 1C). Additionally, we tested whether knockdown of Arid1b expression protected MEFs against senescence induced by oncogenic RAS. While MEFs expressing RAS^G12V^ underwent premature senescence, MEFs infected with shRNAs targeting Arid1b or p53 continued to proliferate (Supplemental Fig. S1E). Moreover, the cells grew anchorage-independently, as assessed by soft agar assay, indicating oncogenic cooperation between RAS^G12V^ and Arid1b knockdown (Fig. 1D). Altogether, these results suggest that knockdown of Arid1b enables bypass of senescence in MEFs.

### Knockdown of Arid1b cooperates in HCC generation by blunting OIS

**Figure 2.**
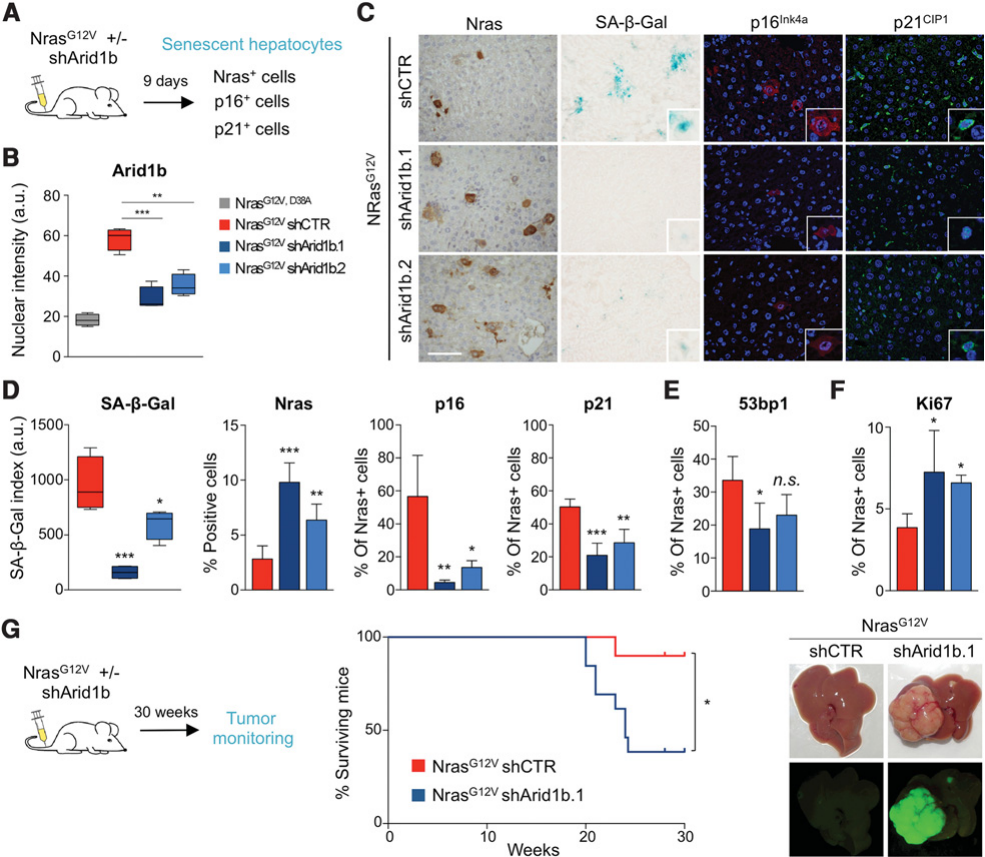
Knockdown of *Arid1b* alleviates OIS in vivo and cooperates in HCC formation. (*A*) Scheme of the in vivo senescence experiments. Transposon constructs carrying *Nras*^*G12V*^ cDNA and Arid1b shRNAs (or control) were delivered into mouse livers by hydrodynamic injection (Nras^G12V/D38A^ was used as negative control). *n* = 4 mice per condition were used. (*B*) Quantification of Arid1b nuclear IF staining in Nras^+^ mouse hepatocytes in the above-described conditions is shown. (*C*) Representative pictures of immunohistochemistry (IHC) for Nras, SA-β-Gal staining, and IF staining for p21^CIP1^ and p16^INK4a^ on liver sections from mice injected with the indicated vectors. (*D*–*F*) Quantification of the SA-β-Gal staining and percentages of Nras-positive, p16^INK4a^-positive, or p21^CIP1^-positive cells (*D*) and 53bp1-positive (*E*) or Ki67-positive (*F*) cells in liver sections from mice injected with the indicated vectors. Graphs represent mean ± SD from *n* = 4 mice. (***) *P* < 0.001; (**) *P* < 0.01; (*) *P* < 0.05; (n.s.) nonsignificant by two-tailed Student *t*-test. (*G*, *left*) Scheme of the in vivo tumor growth experiment. Transposon constructs carrying *Nras*^*G12V*^ cDNA and an Arid1b shRNA (or control) were delivered into mouse livers by hydrodynamic injection. Mice were monitored for 30 wk. *n* = 10 shCTR and *n* = 13 shArid1b.1 mice were used. Kaplan-Meier survival curves (*middle*) and representative pictures of livers (*right*) showing GFP-positive tumor nodules as they were derived from the injected constructs. (*) *P* < 0.05 by log rank (Mantel-Cox) test. Bar, 100 μm. DAPI was used to visualize the nuclei.

Next, we investigated whether Arid1b depletion can prevent senescence in vivo and in this way contribute to liver cancer development. To test this, we used a mouse model in which OIS is triggered in hepatocytes via transposon-mediated transfer of oncogenic Nras (Nras^G12V^) delivered by hydrodynamic tail vein (HDTV) injection (Fig. 2A; Kang et al. 2011). In this model, the senescent hepatocytes activate an immune response that results in their gradual elimination (Kang et al. 2011). To investigate whether Arid1b knockdown affects OIS in vivo, we coexpressed Nras^G12V^ and either control or Arid1b targeting shRNAs. We measured Arid1b levels in Nras^G12V^-expressing hepatocytes 9 d after transposon injection. Hepatocytes expressing an inactive variant (Nras^G12V,D38A^) displayed lower levels of Arid1b than those carrying the constitutively active variant (Nras^G12V^), indicating that oncogenic Nras^G12V^ induces Arid1b expression during senescence in vivo. Furthermore, both shRNAs targeting Arid1b blunted its induction (Fig. 2B; Supplemental Fig. S2A,B). We next evaluated the consequences of depleting Arid1b in Nras-expressing hepatocytes. At day 9, there were more Nras-positive hepatocytes present in the livers of mice transduced with Nras^G12V^_shArid1b than Nras^G12V^_ shCTR vectors (Fig. 2C,D). The higher percentage of Nras^+^ hepatocytes correlated with reduced senescence, as shown by decreased levels of SA-β-Gal in the livers of mice transduced with Nras^G12V^_shArid1b vectors. In addition, a lower percentage of Nras-positive hepatocytes were also positive for p21^Cip1a^ or p16^Ink4a^ upon Arid1b depletion (Fig. 2C,D). Other SWI/SNF components have been suggested to control senescence by regulating p21^Cip1a^ or p16^Ink4a^ transcription (Chai et al. 2005). Interestingly, we also observed a decreased DNA damage response (DDR; assessed by 53BP1 staining) in Nras^+^ hepatocytes of mice transduced with Nras^G12V^_shArid1b vectors (Fig. 2E; Supplemental Fig. S2C), suggesting that there might be additional mechanisms by which Arid1b and, by extension, the SWI/SNF complex control senescence.

Nras^+^ hepatocytes in which Arid1b expression was knocked down also proliferated more, displaying a higher percentage of Ki67^+^ cells (Fig. 2F; Supplemental Fig. S2D). Since depletion of Arid1b blunted the senescence response in vivo and resulted in higher percentages of proliferating Nras^+^ hepatocytes, we investigated whether the knockdown of Arid1b cooperated with Nras^G12V^ in tumor formation. To test this, we used a protocol similar to that used before and monitored the mice regularly for tumor formation (Fig. 2G, left). While only one out of 10 mice injected with the Nras^G12V^_shCTR vector died of tumors during the time they were monitored, eight out of 13 of those bearing the Nras^G12V^_shArid1b.1 construct succumbed to HCCs (Fig. 2G, middle). Fluorescence imaging confirmed that these tumors derived from hepatocytes expressing the transduced vectors, as they were GFP-positive (Fig. 2G, right). Altogether, these results indicate that knockdown of Arid1b contributes to HCC formation by blunting senescence.

### ARID1B regulates OIS in human fibroblasts

**Figure 3.**
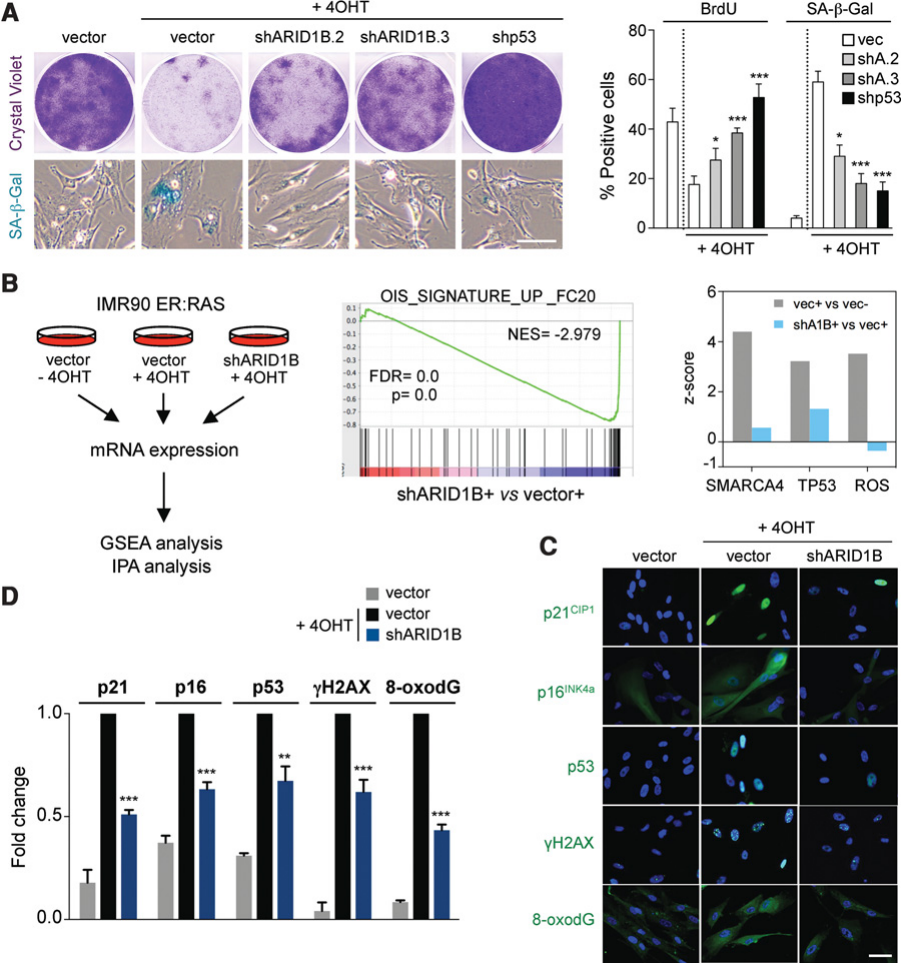
ARID1B regulates OIS in human cells. (*A*) IMR90 ER:RAS cells were transduced with either an empty vector (vec), a p53 shRNA (shp53), or ARID1B shRNAs (shARID1B.2 [shA2] and shARID1B.3 [shA3]) and induced to undergo senescence by 4OHT treatment. Cell proliferation was assessed by crystal violet staining (*top left*) and BrdU incorporation (*right*). Senescence was measured by SA-β-Gal staining (representative pictures are at the *bottom left*, and quantification is at the *right*). (*B*, *left*) IMR90 ER:RAS cells transduced with an empty vector (±4OHT) or ARID1B shRNAs (+4OHT) were subjected to global mRNA expression analysis. Data were subjected to GSEA and IPA. *n* = 3 biological replicates were used. (*Middle*) GSEA showing loss of a signature associated with OIS in the gene expression profile of IMR90 ER:RAS shARID1B versus empty vector, both 4OHT-treated. (NES) Normalized enriched score; (FDR) false discovery rate. (*Right*) IPA upstream regulator analysis of the indicated comparisons is shown. The predicted activation (*z*-score) of SMARCA4, TP53, and reactive oxygen species (ROS) pathways is shown. (*C*,*D*) Quantification by HCA (*D*) of IF staining (*C*) for p21^CIP1^, p16^INK4a^, p53, γH2AX, and 8-oxodG in IMR90 ER:RAS cells transduced with either an empty vector or an shRNA targeting ARID1B (±4OHT). DAPI was used to visualize the nuclei. Graphs represent mean ± SD from *n* = 4 (*A*, BrdU) and *n* = 3 (*A* [SA-β-Gal], *D*). (***) *P* < 0.001; (**) *P* < 0.01; (*) *P* < 0.05 by two-tailed Student *t*-test. Bars, 30 μm.

To investigate the mechanism by which ARID1B controls senescence in more detail, we took advantage of IMR90 human fibroblasts, as they are a widely used system to study senescence and are amenable to mechanistic dissection. First, we checked whether ARID1B is necessary for the implementation of senescence in human cells. To this end, we generated two shRNAs to target human ARID1B (Supplemental Fig. S3A,B) and used them to infect IMR90 human fibroblasts expressing ER:RAS (IMR90 ER:RAS), an inducible model of OIS. Depletion of ARID1B in IMR90 ER:RAS cells blunted OIS, as evidenced by increased proliferation and a decrease in the percentage of cells positive for SA-β-Gal (Fig. 3A). To investigate how the depletion of ARID1B affects OIS, we performed transcriptional profiling of IMR90 ER:RAS cells infected with an shRNA targeting ARID1B (Fig. 3B, left). Using gene set enrichment analysis (GSEA), a signature of OIS was found significantly down-regulated upon ARID1B knockdown (Fig. 3B, middle). GSEA and Ingenuity Pathway Analysis (IPA) showed that SWI/SNF (SMARCA4) target genes were induced during OIS, and this induction was blunted by ARID1B knockdown (Fig. 3B, right; data not shown). Interestingly, IPA also suggested that ARID1B depletion prevented the activation of the p53 pathway and oxidative stress observed during OIS (Fig. 3B, right). Key effectors of senescence such as p16^INK4a^ and p21^CIP1^ were down-regulated at both the mRNA and protein level upon ARID1B depletion (Fig. 3C,D; Supplemental Fig. S3C). Consistent with the observations in vivo, depletion of ARID1B resulted in reduced DDR (Fig. 3C,D). In addition, confirming the IPA, ARID1B knockdown resulted in decreased levels of p53 and oxidative stress (as measured by 8-oxodG staining) (Fig. 3C,D).

### ARID1B expression triggers a senescent-like arrest

**Figure 4.**
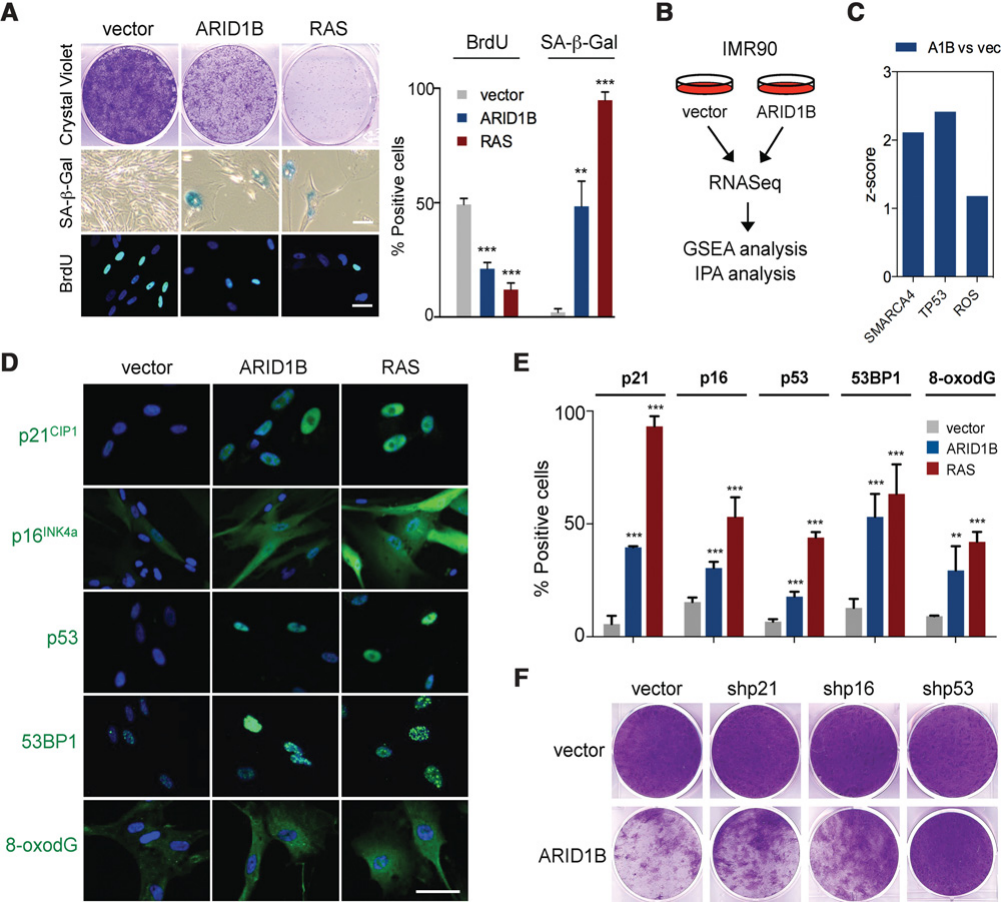
Ectopic expression of ARID1B induces senescence. (*A*,*D*,*E*) IMR90 cells were transduced with either an empty vector, *ARID1B* cDNA, or *Ras*^*G12V*^ cDNA and then analyzed for cell growth (crystal violet), SA-β-Gal activity, and BrdU incorporation (*A*) as well as IF staining (*D*) using the indicated antibodies. DAPI was used to visualize the nuclei. (*E*) Quantification by HCA of IF staining in *D*. Data represent mean ± SD from *n* = 3 (*A*, SA-β-Gal) and *n* = 4 (*A* [BrdU], *E*). (*B*) RNA sequencing (RNA-seq) was performed with IMR90 cells transduced with either an empty vector or *ARID1B* cDNA. Data were subjected to GSEA and IPA. *n* = 3 biological replicates were used. (*C*) IPA upstream regulator analysis of the RNA-seq reveals activation (*z*-score) of SMARCA4, TP53, and ROS pathways. (*F*) IMR90 cells were cotransduced with an empty vector or *ARID1B* cDNA and shRNAs targeting p21^CIP1^, p16^INK4a^, or p53. Cell proliferation was assessed by crystal violet staining. (***) *P* < 0.001; (**) *P* < 0.01 by two-tailed Student *t*-test. Bars, 30 μm.

To complement the knockdown studies, we analyzed the effect of expressing ARID1B in IMR90 cells. Expression of ARID1B in IMR90 cells induced growth arrest, SA-β-Gal activity, and a decrease in the percentage of BrdU-positive cells (Fig. 4A; Supplemental Fig. S4A). By analyzing the transcriptional profiles of IMR90 cells infected with a control vector or ARID1B by GSEA, we observed that ARID1B overexpression was associated with signatures of OIS and senescence-related phenotypes such as SASP and cell cycle arrest (Supplemental Fig. S4B). IPA confirmed that ARID1B expression resulted in the up-regulation of SWI/SNF target genes and the induction of the DDR and p53 (Fig. 4B,C). Indeed, the expression of ARID1B not only induced p16^INK4a^ and p21^CIP1a^ but also up-regulated p53 levels and caused DNA damage and oxidative stress (Fig. 4D,E). We then interrogated the relative contribution of these pathways to the ARID1B-mediated arrest using shRNAs. The arrest caused by ARID1B was prevented partially when the expression of p16^INK4a^ or p21^CIP1a^ had been knocked down (Fig. 4F; Supplemental Fig. S4C). Interestingly, knocking down p53 prevented to a larger extent the arrest caused by ARID1B expression (Fig. 4F), suggesting an important role of the p53 pathway in ARID1B-mediated arrest. Altogether, these observations reinforce the idea that, in addition to regulating p16^INK4a^ and p21^CIP1^ transcription, ARID1B uses additional mechanisms, such as the generation of p53-activating stress signals (DDR and oxidative stress), to control senescence.

### Identification of novel ARID1B targets controlling senescence

**Figure 5.**
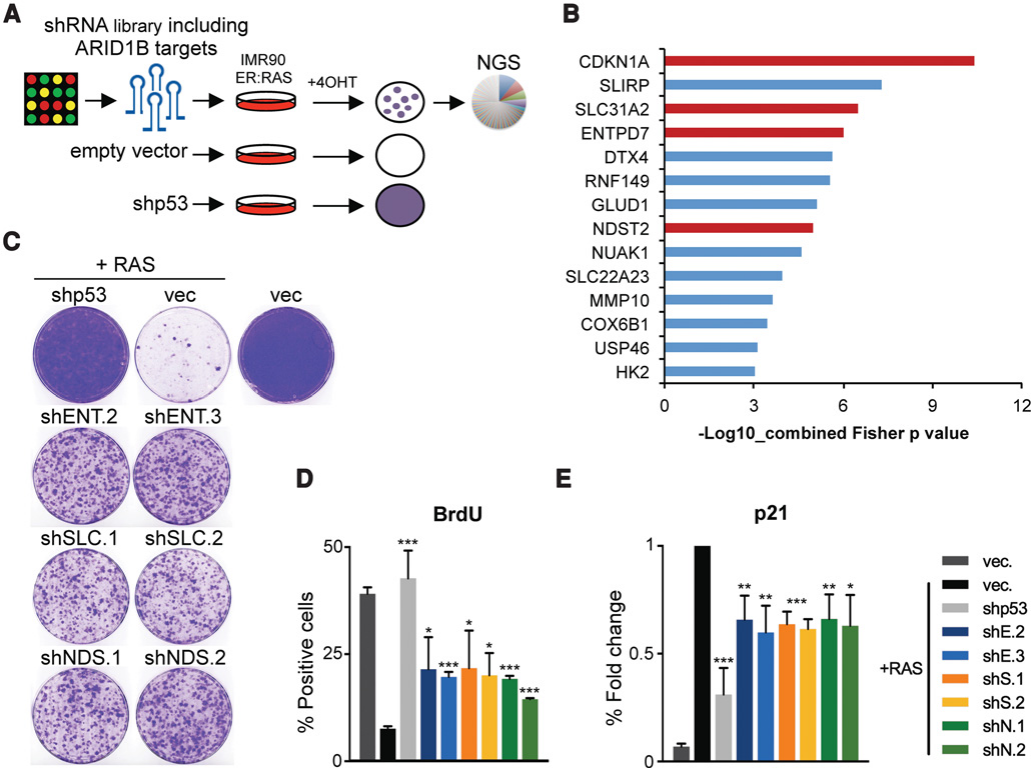
A focused shRNA screen identified novel ARID1B effectors controlling senescence. (*A*) Schematic model of the bypass of senescence screen using a library of shRNAs targeting genes up-regulated in OIS, including some that are ARID1B-dependent. An average of six different shRNAs per gene were used. After infection with the shRNA library or controls, IMR90 ER:RAS cells were treated with 4OHT and passaged until library-infected cells bypassed senescence. shRNAs that bypassed senescence were recovered by NGS. The screen was performed in duplicate. (*B*) List of significantly enriched genes by Fisher's combined *P*-value (*P* < 0.001). *CDKN1A* and genes selected for further analysis are marked in red. (*C*–*E*) Colony formation assay (*C*), BrdU incorporation (*D*), and p21 analysis (*E*) of IMR90 cotransduced with RAS^G12V^ (or empty vector) and shRNAs for ENTPD7 (shE2 and shE3), SLC31A2 (shS1 and shS2), or NDST2 (shN1 and shN2). A p53 shRNA was used as control. Quantifications of IF staining were performed by HCA. *n* = 3. Graphs represents mean ± SD. (***) *P* < 0.001; (**) *P* < 0.005; (*) *P* < 0.05 by two-tailed Student *t*-test.

To identify novel mechanisms by which ARID1B and, by extension, the SWI/SNF complex regulate senescence, we generated an shRNA library targeting 255 genes up-regulated during OIS. It included a subset of ARID1B-dependent genes based on our gene expression analysis (Supplemental Fig. S5A). We designed up to six shRNAs targeting each gene and cloned the library in a miRE-based vector that has improved knockdown efficiency when compared with other shRNA vectors (Fellmann et al. 2013). Using this library, we carried out a screen for bypass of OIS in IMR90 ER:RAS cells (Fig. 5A; Supplemental Fig. S5B). After infection with the library or controls, senescence was induced by adding 4OHT to activate ER:RAS, and cells were passaged until growth (indicative of senescence bypass) was observed in library-infected plates. The distribution of shRNAs at different time points was calculated by NGS. Importantly, by including up to six shRNAs targeting each gene in the library, we were able to analyze the results of the screen at the gene level rather than the shRNA level. Statistical analysis using EdgeR unveiled 14 genes whose shRNAs were significantly enriched (*P* < 0.001) in cells bypassing OIS. Interestingly, 13 of the 14 candidates identified in the screen were ARID1B-dependent genes (Supplemental Fig. S5C). Using IPA, we independently confirmed a significant enrichment for SWI/SNF (SMARCA4) target genes among the top hits of the screen (Supplemental Fig. S5D) and for genes related to p53 and reactive oxygen species (ROS) (Supplemental Fig. S5D). The top gene identified in the screen was *CDKN1A* (Fig. 5B; Supplemental Fig. S5E), which encodes for p21^CIP1^, a key senescence regulator. Our data and the literature (Chai et al. 2005) support an important role for p21^CIP1a^ in implementing SWI/SNF-mediated senescence. This served as an internal control and confirmed the validity of our approach. Among the other genes identified, at least three (*SLC31A2*, *ENTPD7*, and *NDST2*) could be functionally associated with the generation of oxidative stress or DDR. We confirmed that these three genes were SWI/SNF targets induced during OIS (Supplemental Fig. S5F–H). To investigate the role of SLC31A2, NDST2, and ENTPD7 in OIS, we generated two shRNAs targeting each gene (Supplemental Fig. S6A). Knocking down either ENTPD7, SLC31A2, or NDST2 in IMR90 ER:RAS cells did not affect the strength of RAS/ERK activation (Supplemental Fig. S6B) but prevented the senescent arrest (Fig. 5C,D) and blunted p21^CIP1a^ induction (Fig. 5E). Similarly, depletion of ENTPD7, SLC31A2, or NDST2 partially prevented the senescent arrest caused by ARID1B expression (Supplemental Fig. S6C,D). These results confirm the identification of novel mediators of the effects of ARID1B in OIS.

### Multiple ARID1B target genes mediate induction of OIS in the liver

**Figure 6.**
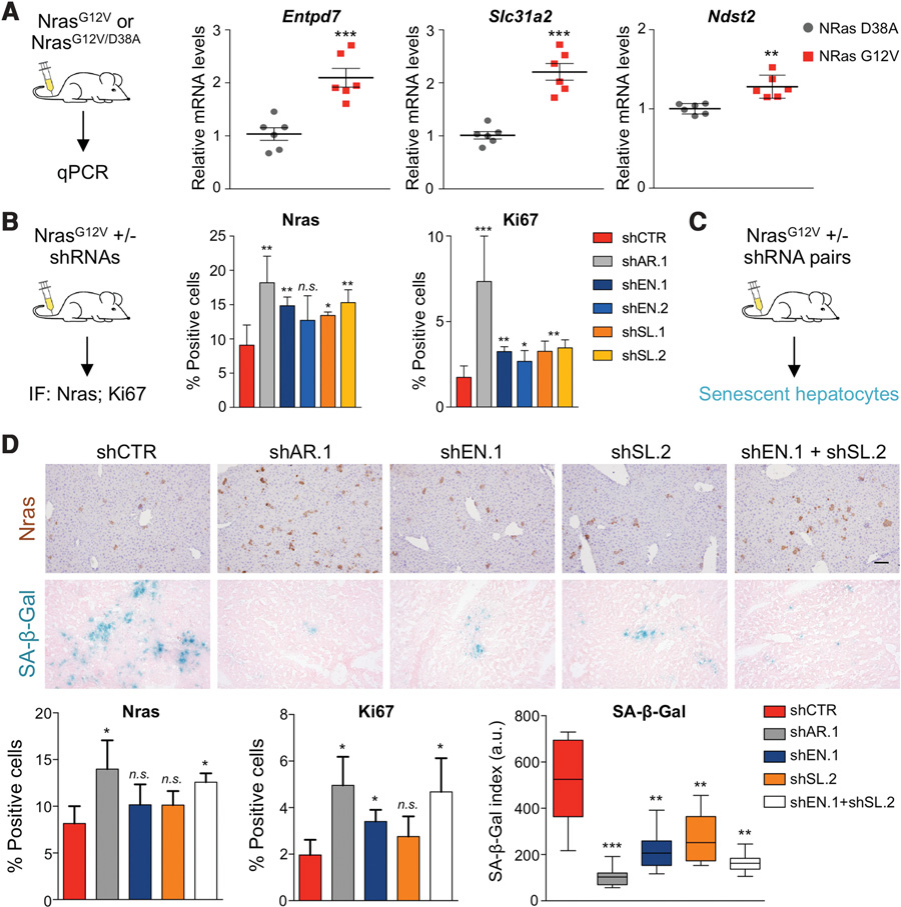
ARID1B controls senescence by coordinated regulation of multiple targets. (*A*) Nras^G12V^ or Nras^G12V/D38A^ transposon constructs were delivered into mouse livers by hydrodynamic injection. Livers were harvested after 6 d, followed by mRNA extraction and quantitative RT–PCR analysis for *Entpd7*, *Slc31a2*, and *Ndst2* mRNA levels. *n* = 6 mice per condition were used. (*B*) Transposon constructs carrying *Nras*^*G12V*^ cDNA and Arid1b shRNAs (shSL.1 and shSL.2) or control (shCTR) were delivered into mouse livers by hydrodynamic injection. *n* = 4 mice per condition were used. Livers were harvested after 6 d, and sections were stained for Nras and Ki67. Quantification of IF staining is shown at the *right*. Graphs represent mean ± SD. (***) *P* < 0.001; (**) *P* < 0.005; (*) *P* < 0.05; (n.s.) nonsignificant by two-tailed Student *t*-test. (*C,D*) Transposon constructs carrying *Nras*^*G12V*^ cDNA and combinations of two control shRNAs, one control and one targeting Arid1b (shAR.1), one control and one targeting Entpd7 (shEN.1), one control and one targeting Slc31a2 (shSL.2), and a combination of Entpd7 and Slc31a2 shRNAs (shEN.1 + shSL.2) were delivered into mouse livers by hydrodynamic injection. In this experiment, a more active transposase (SB100) that allows integration of multiple shRNAs was used. *n* = 3 mice per condition were used. Livers were harvested after 9 d, and sections were stained for Nras, Ki67, and SA-β-Gal. Representative pictures of IHC for Nras and SA-β-Gal staining are shown. Quantification of the staining is shown at the *bottom*. Bar, 100 μm. Graphs represents mean ± SD. (***) *P* < 0.001; (**) *P* < 0.005; (*) *P* < 0.05; (n.s.) nonsignificant by two-tailed Student *t*-test.

We next analyzed whether the ARID1B targets identified in the screen have a role in mediating OIS in vivo. First, we tested whether they were induced in livers transduced with oncogenic Nras (Nras^G12V^). We observed an increase in *Entpd7*, *Slc31a2*, and *Ndst2* expression by Nras^G12V^ in an Arid1b-dependent fashion (Fig. 6A; Supplemental Fig. S7A), suggesting that the coordinated regulation of multiple SWI/SNF-dependent genes could mediate the effects of Arid1b on senescence.

To test this, we examined whether Entpd7 and Slc31a2 mediate OIS in vivo. We focused on these two genes because they are down-regulated in a large subset of human HCCs (Supplemental Fig. S7B), and their depletion blunted senescence strongly in vitro. We transduced mice with vectors coexpressing oncogenic Nras (Nras^G12V^) and a control shRNA or shRNAs targeting Arid1b, Entpd7, or Slc31a2 (Fig. 6B, left). Knockdown of Entpd7 or Slc31a2 resulted in increased numbers of Nras^+^ hepatocytes 6 d after transduction (Fig. 6B, middle; Supplemental Fig. S7C). Moreover, knocking down Entpd7 or Slc31a2 led to an increase in the proportion of Nras^+^ hepatocytes that were proliferating, suggesting bypass of senescence (Fig. 6B, right). However, the effects observed upon depletion of either Entpd7 or Slc31a2 were not as pronounced as those observed upon Arid1b depletion (Fig. 6B), perhaps because Arid1b regulates multiple targets to control senescence.

To explore this hypothesis, we devised a modified version of the HDTV injection assay that combined transduction of two transposon vectors with the more active SB100 transposase to make possible the simultaneous down-regulation of two genes (Fig. 6C). We tested the effect that the combined knockdown of Entpd7 and Slc31a2 had on OIS of hepatocytes in vivo by comparing the results with knockdown of Arid1b (Fig. 6D; Supplemental Fig. S7D). In this assay, individual knockdown of Entpd7 or Slc31a2 caused a modest increase in the percentage of Nras^+^ hepatocytes present 9 d after transduction. There was a clearer increase in the percentage of proliferating Ki67^+^/Nras^+^ hepatocytes upon individual knockdown of Entpd7 or Slc31a2, but it was not comparable with that observed upon Arid1b knockdown in the same setting (Fig. 6D). Interestingly, the combined depletion of Entpd7 and Slc31a2 resulted in an increased proportion of Nras^+^ and Ki67^+^/Nras^+^ hepatocytes, comparable with that observed upon Arid1b knockdown (Fig. 6D). A similar trend was observed when analyzing senescence by SA-β-Gal staining. The combined knockdown of Entpd7 and Slc31a2 resulted in a significant decrease of senescence similar to that achieved upon Arid1b down-regulation (Fig. 6D). Overall, these results confirm that ARID1B controls senescence by coordinating the regulation of multiple targets.

### Nucleotide metabolism is a potential liability of ARID1B mutant tumors

**Figure 7.**
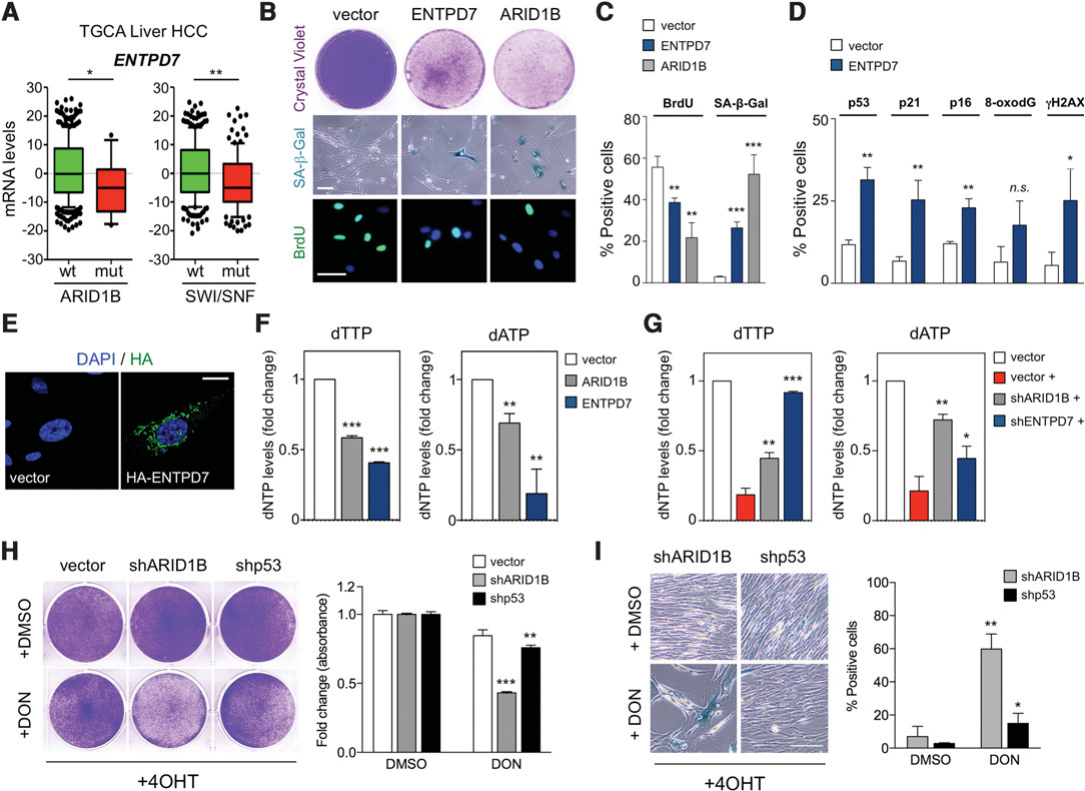
ARID1B-deficient cells are sensitive to alterations in nucleotide metabolism. (*A*) ENTPD7 differential expression between SWI/SNF or ARID1B mutant/wild-type samples was calculated using Student's *t*-test (details are in the Materials and Methods) using The Cancer Genome Atlas Liver Hepatocellular Carcinoma data set. (*B*) Colony formation assay (*top*), SA-β-Gal staining (*middle*), and BrdU IF (*bottom*) of IMR90 transduced with an empty vector or cDNA of *ARID1B* or *ENTPD7*. Bar, 30 μm. (*C*) Quantification by HCA of BrdU IF and SA-β-Gal staining is shown. *n* = 3. (*D*) Quantification by HCA of IF staining for p53, p21^CIP1^, p16^INK4a^, 8-oxodG, and γH2AX in IMR90 cells transduced with *ENTPD7* cDNA or empty vector. *n* = 3. (*E*) Representative images of HA tag IF staining of IMR90 cells transduced with either an empty vector or an HA-tagged *ENTPD7* cDNA. DAPI was used to visualize the nuclei. Bar, 10 μm. (*F*) IMR90 cells were transduced with either an empty vector, *ARID1B* cDNA, or *ENTPD7* cDNA, and then intracellular dNTP levels were measured. (*G*) IMR90 ER:RAS cells were transduced with either an empty vector, an ARID1B shRNA, or an ENTPD7 shRNA and induced to undergo senescence by 4OHT treatment (+). Intracellular dNTP levels were measured 5 d after 4OHT. (*H*,*I*) Cell growth assay (*H*, crystal violet) and SA-β-Gal staining (*I*) of stable ARID1B knockdown, p53 knockdown, or control IMR90 ER:RAS cells treated with 4OHT followed by 5 µM 6-diazo-5-oxo-L-norleucine (DON) or DMSO. For *H* and *I*, the *left* panels show representative images, and the *right* panels show quantification. Graphs represent mean ± SD from *n* = 2 (*H*) and *n* = 3 (*F*,*G*,*I*). (***) *P* < 0.001; (**) *P* < 0.005; (*) *P* < 0.05; (n.s.) nonsignificant by two-tailed Student *t*-test.

Given the prominent tumor suppressor role of senescence, strategies aimed to reactivate senescence in tumors (so-called prosenescence therapies) have been explored to target cancer (Nardella et al. 2011; Acosta and Gil 2012). As depletion of ARID1B prevents senescence, we postulated that senescence reactivation could be used to target SWI/SNF mutant tumors. Among the ARID1B targets mediating senescence, ENTPD7 was of particular relevance, as it is an enzyme involved in nucleotide catabolism (Shi et al. 2001). Shortage of deoxyribonucleotides (dNTPs) has been shown previously to cause OIS via activation of DDR (Aird et al. 2013). Interestingly, a significant correlation exists between the expression levels of ENTPD7 and ARID1B in several cancer types, including liver cancer (Supplemental Fig. S8A,B). Moreover, when HCC cases were stratified according to the mutational status of ARID1B or the SWI/SNF complex, we observed significantly lower ENTPD7 expression in the mutated samples (Fig. 7A).

Ectopic expression of ENTPD7 in IMR90 cells caused growth arrest and senescence (Fig. 7B,C). ENTPD7 expression induced several senescent effectors—including p16^INK4a^ but more significantly p53 and p21^CIP1a^—and increased DNA damage (Fig. 7D). ENTPD7 belongs to the ENTPD family of ectonucleoside triphosphate diphosphohydrolases. Despite the name, membrane topology analysis predicts that some ENTPDs, such as ENTPD5 and ENTPD7, will reside inside cells and not at their surface (Robson et al. 2006). Therefore, these ENTPDs could affect the intracellular pools of nucleotides, as shown previously for ENTPD5 (Fang et al. 2010).

To investigate the localization of ENTPD7, given the lack of reliable antibodies, we generated an HA-tagged ENTPD7 version. ENTPD7 localized intracellularly to the Golgi apparatus (Fig. 7E; Supplemental Fig. S8D), in contrast to ENTPD5, which has been described as residing in the ER (Fang et al. 2010). In agreement with its localization, ENTPD7 expression resulted in decreased intracellular dNTP levels in IMR90 cells (Fig. 7F; Supplemental Fig. S8E). Conversely, ENTPD7 knockdown prevented the decrease of dNTPs levels observed during OIS (Fig. 7G; Supplemental Fig. S8F). Interestingly, ectopic expression of ARID1B also affected dNTP levels (particularly dTTP and dATP) in a manner similar to ENTPD7 (Fig. 7F,G; Supplemental Fig. S8E,F). Consistent with this, IPA suggested an association between ARID1B expression and pyrimidine metabolism (Supplemental Fig. S8C).

Based on these observations, we decided to test whether nucleotide metabolism could constitute a liability of ARID1B-deficient cells and potentially of SWI/SNF mutated tumors (Supplemental Fig. S9A). We expressed ENTPD7 in IMR90 ER:RAS cells infected with a control vector or shRNAs targeting ARID1B or p53 (Supplemental Fig. S9B). Expression of ENTPD7 significantly affected the growth of ARID1B-depleted but not p53-depleted cells. The experiment suggested that targeting nucleotide metabolism could be a liability of ARID1B-depleted cells. To directly test this possibility, we took advantage of 6-diazo-5-oxo-L-norleucine (DON) and gemcitabine, two drugs inhibiting nucleotide metabolism (Barclay et al. 1962; Wang et al. 2009). Treatment with 5 µM DON did not affect the growth of IMR90 ER:RAS control cells or p53-depleted cells but significantly arrested ARID1B-depleted cells (Fig. 7H). Similar effects were observed when cells were treated with 5 µM gemcitabine (Supplemental Fig. S9C). Importantly, the growth arrest in ARID1B-depleted cells was associated with induction of senescence (Fig. 7I), suggesting that targeting nucleotide metabolism could be used as a prospective prosenescence therapy against ARID1B mutant tumors

## Discussion

Sequencing cancer genomes is providing us with precise information on the recurrent genetic alterations present in different tumor types. Despite this progress, our knowledge of how some of these mutations affect cancer has not advanced at a similar rate due to the lack of functional information. Since bypass of senescence is a common feature of cancers, we interrogated the ability of genes frequently deleted in HCC to regulate senescence.

In a genetic screen, we identified the SWI/SNF component ARID1B as a regulator of senescence. Other members of the SWI/SNF complex have been linked previously with senescence, including SNF5, BRD7, and BRG1 (Oruetxebarria et al. 2004; Chai et al. 2005; Burrows et al. 2010; Drost et al. 2010). Interestingly, ablation of ARID1B in vivo prevents OIS, results in increased proliferation of hepatocytes expressing oncogenic Nras^G12V^, and cooperates in oncogenic transformation. Regulation of p16^INK4a^ and p21^CIP1^ are two suggested mechanisms by which SWI/SNF complexes control senescence (Oruetxebarria et al. 2004; Chai et al. 2005). In addition to down-regulating the expression of both CDK inhibitors, manipulating ARID1B levels also affects oxidative stress and DDR. Taking this into consideration, we screened for additional ARID1B targets mediating senescence. Our results suggest that SWI/SNF induces senescence not only by controlling p16^INK4a^ and p21^CIP1^ expression but also via the coordinated regulation of other genes, including ENTPD7, SLC31A2, and NDST2. Notably, these three novel regulators of senescence identified here have in common that, by affecting DDR or oxidative stress, they could contribute to the activation of the p53/p21 axis.

Due to the high frequency of mutations on SWI/SNF components, there is a strong interest in developing therapies to target these cancers. Studies looking into vulnerabilities of ARID1A and BRG1 mutant cancers have proposed to target residual SWI/SNF complexes in what has been termed “cancer-selective paralog dependency” (Helming et al. 2014b; Hoffman et al. 2014). Other therapies for ARID1A-mutated cancers include inhibition of EZH2 (Bitler et al. 2015), PI3K (Samartzis et al. 2014; Bitler et al. 2015), or PARP (Shen et al. 2015). However, there is still the need to identify therapies that could target the whole spectrum of SWI/SNF mutant tumors. Recently, treatments that invoke a senescence response (so-called prosenescence therapies) have been tested against several tumor types (Nardella et al. 2011; Acosta and Gil 2012). Since senescence is central to the tumor suppressor properties of SWI/SNF, we took advantage of the gained mechanistic insights explaining how ARID1B regulates senescence to target ARID1B-depleted cells. Among the ARID1B targets, we singled out ENTPD7, an enzyme involved in nucleotide metabolism. Nucleotide metabolism has been linked previously with DDR activation and ultimately OIS (Aird et al. 2013). Cells with reduced ARID1B expression underwent a strong senescence arrest in response to ENTPD7 expression or treatment with inhibitors of nucleotide metabolism (Fig. 7). It could be argued that this response would imply intact p53 signaling. Interestingly, some studies have highlighted mutual exclusivity between SWI/SNF and p53 mutations (Guan et al. 2011; Kadoch et al. 2013). Although the connection between SWI/SNF and p53 is complex, our results suggest a functional overlap between both pathways that could explain their mutual exclusivity in cancer (Supplemental Fig. S9B). In turn, the wild-type p53 status of a significant subset of SWI/SNF mutant tumors will make it more sensitive to nucleotide metabolism inhibitors.

In conclusion, we identified *ARID1B* as a gene commonly altered in HCC with the ability to control senescence. Regulation of senescence could explain in part the role of SWI/SNF in tumor suppression. By unveiling novel pathways regulated by SWI/SNF in senescence, our investigation suggests novel therapeutic targets, such as nucleotide metabolism, that could be relevant to treat tumors deficient in ARID1B and, by extension, other SWI/SNF components.

## Materials and methods

### Cell culture and retroviral and lentiviral infection

HEK-293T and IMR90 cells were obtained from American Type Culture Collection. MEFs were prepared as described in the Supplemental Material. Cell lines were grown and infected with retroviral or lentiviral vectors as described in the Supplemental Material.

### BrdU incorporation, crystal violet staining, and SA-β-gal staining

BrdU incorporation, crystal violet staining, and SA-β-gal staining have been described previously (Banito et al. 2009; Barradas et al. 2009; Debacq-Chainiaux et al. 2009; Acosta et al. 2013).

### Soft agar assay

Ras^V12G^-infected MEFs were suspended in complete DMEM containing 0.35% agar (Sigma), seeded in triplicate on six-well plates precoated with 0.7% agar in complete growth medium, and incubated at 37°C and 5% CO_2_. After 21 d, colonies were photographed and counted in 10 randomly chosen fields.

### Gene expression analysis

Total RNA was extracted using Trizol reagent (Invitrogen) and the RNeasy isolation kit (Qiagen). cDNAs were generated using SuperScript II reverse transcriptase (Invitrogen), dNTPs, and random hexamers. PCR reactions were performed in a real-time PCR detection system (Bio-Rad) using Power SYBR Green master mix or TaqMan universal PCR master mix (Applied Biosystems). Expression was normalized to ribosomal protein S14 (*RPS14*) expression. The primer sets used are in Supplemental Table S3. Mouse tissue samples were weighed and ground to a fine powder in liquid nitrogen. The RNA was then extracted and processed as described above.

### In vivo senescence and tumorigenesis experiments

For all animal experiments, only mice in a C57BL/6 background were used (equal gender distribution in randomized groups). C57BL/6 mice were purchased from Charles River. At day 0, HDTV injection of a transposon-based Nras expression plasmid together with an expression plasmid for sleeping beauty 13 was performed, as described in detail previously (Kang et al. 2011). Animals were sacrificed, and livers were collected at specific time points (days 6 and 9) or, in the tumorigenesis study, when termination criteria were fulfilled. Samples were fixed and subjected to immunohistochemistry analysis as described in the Supplemental Material. The antibodies used are in Supplemental Table S4. No statistical method was used to predetermine sample size. The investigators were not blinded to allocation during experiments and outcome assessment. All mice were maintained under specific pathogen-free conditions in accordance with the institutional guidelines of the University of Tübingen. The German legal authorities approved these experiments.

### Immunofluorescence (IF) and high-content analysis (HCA)

IF was performed as described previously (Banito et al. 2009) using the antibodies in Supplemental Table S4. Details of HCA, IF in mouse tissue, and confocal microscopy are in the Supplemental Material.

### Statistical analysis

All statistical analyses were performed by two-tailed Student *t*-test unless stated otherwise.

## Supplementary Material

Supplemental Material

## References

[TORDELLAGAD286112C1] Acosta JC, Gil J. 2012 Senescence: a new weapon for cancer therapy. Trends Cell Biol 22: 211–219. 22245068 10.1016/j.tcb.2011.11.006

[TORDELLAGAD286112C2] Acosta JC, Banito A, Wuestefeld T, Georgilis A, Janich P, Morton JP, Athineos D, Kang TW, Lasitschka F, Andrulis M, 2013 A complex secretory program orchestrated by the inflammasome controls paracrine senescence. Nat Cell Biol 15: 978–990. 23770676 10.1038/ncb2784 PMC3732483

[TORDELLAGAD286112C3] Aird KM, Zhang G, Li H, Tu Z, Bitler BG, Garipov A, Wu H, Wei Z, Wagner SN, Herlyn M, 2013 Suppression of nucleotide metabolism underlies the establishment and maintenance of oncogene-induced senescence. Cell Rep 3: 1252–1265. 23562156 10.1016/j.celrep.2013.03.004 PMC3840499

[TORDELLAGAD286112C4] Banito A, Rashid ST, Acosta JC, Li S, Pereira CF, Geti I, Pinho S, Silva JC, Azuara V, Walsh M, 2009 Senescence impairs successful reprogramming to pluripotent stem cells. Genes Dev 23: 2134–2139. 19696146 10.1101/gad.1811609 PMC2751980

[TORDELLAGAD286112C5] Barclay RK, Garfinkel E, Phillipps MA. 1962 Effects of 6-diazo-5-oxo-L-norleucine on the incorporation of precursors into nucleic acids. Cancer Res 22: 908–914. 13864924

[TORDELLAGAD286112C6] Barradas M, Anderton E, Acosta JC, Li S, Banito A, Rodriguez-Niedenfuhr M, Maertens G, Banck M, Zhou MM, Walsh MJ, 2009 Histone demethylase JMJD3 contributes to epigenetic control of INK4a/ARF by oncogenic RAS. Genes Dev 23: 1177–1182. 19451218 10.1101/gad.511109 PMC2685533

[TORDELLAGAD286112C7] Bitler BG, Aird KM, Garipov A, Li H, Amatangelo M, Kossenkov AV, Schultz DC, Liu Q, Shih Ie M, Conejo-Garcia JR, 2015 Synthetic lethality by targeting EZH2 methyltransferase activity in ARID1A-mutated cancers. Nat Med 21: 231–238. 25686104 10.1038/nm.3799 PMC4352133

[TORDELLAGAD286112C8] Burrows AE, Smogorzewska A, Elledge SJ. 2010 Polybromo-associated BRG1-associated factor components BRD7 and BAF180 are critical regulators of p53 required for induction of replicative senescence. Proc Natl Acad Sci 107: 14280–14285. 20660729 10.1073/pnas.1009559107 PMC2922604

[TORDELLAGAD286112C9] Chai J, Charboneau AL, Betz BL, Weissman BE. 2005 Loss of the hSNF5 gene concomitantly inactivates p21CIP/WAF1 and p16INK4a activity associated with replicative senescence in A204 rhabdoid tumor cells. Cancer Res 65: 10192–10198. 16288006 10.1158/0008-5472.CAN-05-1896

[TORDELLAGAD286112C10] Cheng SW, Davies KP, Yung E, Beltran RJ, Yu J, Kalpana GV. 1999 c-MYC interacts with INI1/hSNF5 and requires the SWI/SNF complex for transactivation function. Nat Genet 22: 102–105. 10319872 10.1038/8811

[TORDELLAGAD286112C11] Collado M, Serrano M. 2010 Senescence in tumours: evidence from mice and humans. Nat Rev Cancer 10: 51–57. 20029423 10.1038/nrc2772 PMC3672965

[TORDELLAGAD286112C12] Debacq-Chainiaux F, Erusalimsky JD, Campisi J, Toussaint O. 2009 Protocols to detect senescence-associated β-galactosidase (SA-βgal) activity, a biomarker of senescent cells in culture and in vivo. Nat Protoc 4: 1798–1806. 20010931 10.1038/nprot.2009.191

[TORDELLAGAD286112C13] Drost J, Mantovani F, Tocco F, Elkon R, Comel A, Holstege H, Kerkhoven R, Jonkers J, Voorhoeve PM, Agami R, 2010 BRD7 is a candidate tumour suppressor gene required for p53 function. Nat Cell Biol 12: 380–389. 20228809 10.1038/ncb2038

[TORDELLAGAD286112C14] Fang M, Shen Z, Huang S, Zhao L, Chen S, Mak TW, Wang X. 2010 The ER UDPase ENTPD5 promotes protein N-glycosylation, the Warburg effect, and proliferation in the PTEN pathway. Cell 143: 711–724. 21074248 10.1016/j.cell.2010.10.010

[TORDELLAGAD286112C15] Fellmann C, Hoffmann T, Sridhar V, Hopfgartner B, Muhar M, Roth M, Lai DY, Barbosa IA, Kwon JS, Guan Y, 2013 An optimized microRNA backbone for effective single-copy RNAi. Cell Rep 5: 1704–1713. 24332856 10.1016/j.celrep.2013.11.020

[TORDELLAGAD286112C16] Fujimoto A, Totoki Y, Abe T, Boroevich KA, Hosoda F, Nguyen HH, Aoki M, Hosono N, Kubo M, Miya F, 2012 Whole-genome sequencing of liver cancers identifies etiological influences on mutation patterns and recurrent mutations in chromatin regulators. Nat Genet 44: 760–764. 22634756 10.1038/ng.2291

[TORDELLAGAD286112C17] Guan B, Wang TL, Shih Ie M. 2011 ARID1A, a factor that promotes formation of SWI/SNF-mediated chromatin remodeling, is a tumor suppressor in gynecologic cancers. Cancer Res 71: 6718–6727. 21900401 10.1158/0008-5472.CAN-11-1562 PMC3206175

[TORDELLAGAD286112C18] Hanahan D, Weinberg RA. 2011 Hallmarks of cancer: the next generation. Cell 144: 646–674. 21376230 10.1016/j.cell.2011.02.013

[TORDELLAGAD286112C19] Helming KC, Wang X, Roberts CW. 2014a Vulnerabilities of mutant SWI/SNF complexes in cancer. Cancer Cell 26: 309–317. 25203320 10.1016/j.ccr.2014.07.018 PMC4159614

[TORDELLAGAD286112C20] Helming KC, Wang X, Wilson BG, Vazquez F, Haswell JR, Manchester HE, Kim Y, Kryukov GV, Ghandi M, Aguirre AJ, 2014b ARID1B is a specific vulnerability in ARID1A-mutant cancers. Nat Med 20: 251–254. 24562383 10.1038/nm.3480 PMC3954704

[TORDELLAGAD286112C21] Hoffman GR, Rahal R, Buxton F, Xiang K, McAllister G, Frias E, Bagdasarian L, Huber J, Lindeman A, Chen D, 2014 Functional epigenetics approach identifies BRM/SMARCA2 as a critical synthetic lethal target in BRG1-deficient cancers. Proc Natl Acad Sci 111: 3128–3133. 24520176 10.1073/pnas.1316793111 PMC3939885

[TORDELLAGAD286112C22] Jacobs JJ, Keblusek P, Robanus-Maandag E, Kristel P, Lingbeek M, Nederlof PM, van Welsem T, van de Vijver MJ, Koh EY, Daley GQ, 2000 Senescence bypass screen identifies TBX2, which represses Cdkn2a (p19ARF) and is amplified in a subset of human breast cancers. Nat Genet 26: 291–299. 11062467 10.1038/81583

[TORDELLAGAD286112C23] Kadoch C, Hargreaves DC, Hodges C, Elias L, Ho L, Ranish J, Crabtree GR. 2013 Proteomic and bioinformatic analysis of mammalian SWI/SNF complexes identifies extensive roles in human malignancy. Nat Genet 45: 592–601. 23644491 10.1038/ng.2628 PMC3667980

[TORDELLAGAD286112C24] Kang TW, Yevsa T, Woller N, Hoenicke L, Wuestefeld T, Dauch D, Hohmeyer A, Gereke M, Rudalska R, Potapova A, 2011 Senescence surveillance of pre-malignant hepatocytes limits liver cancer development. Nature 479: 547–551. 22080947 10.1038/nature10599

[TORDELLAGAD286112C25] Khursheed M, Kolla JN, Kotapalli V, Gupta N, Gowrishankar S, Uppin SG, Sastry RA, Koganti S, Sundaram C, Pollack JR, 2013 ARID1B, a member of the human SWI/SNF chromatin remodeling complex, exhibits tumour-suppressor activities in pancreatic cancer cell lines. Br J Cancer 108: 2056–2062. 23660946 10.1038/bjc.2013.200 PMC3670478

[TORDELLAGAD286112C26] Kia SK, Gorski MM, Giannakopoulos S, Verrijzer CP. 2008 SWI/SNF mediates polycomb eviction and epigenetic reprogramming of the INK4b–ARF–INK4a locus. Mol Cell Biol 28: 3457–3464. 18332116 10.1128/MCB.02019-07 PMC2423153

[TORDELLAGAD286112C27] Kondoh H, Lleonart ME, Gil J, Wang J, Degan P, Peters G, Martinez D, Carnero A, Beach D. 2005 Glycolytic enzymes can modulate cellular life span. Cancer Res 65: 177–185. 15665293

[TORDELLAGAD286112C28] Kuilman T, Michaloglou C, Mooi WJ, Peeper DS. 2010 The essence of senescence. Genes Dev 24: 2463–2479. 21078816 10.1101/gad.1971610 PMC2975923

[TORDELLAGAD286112C29] Lee D, Kim JW, Seo T, Hwang SG, Choi EJ, Choe J. 2002 SWI/SNF complex interacts with tumor suppressor p53 and is necessary for the activation of p53-mediated transcription. J Biol Chem 277: 22330–22337. 11950834 10.1074/jbc.M111987200

[TORDELLAGAD286112C30] Lee JJ, Sholl LM, Lindeman NI, Granter SR, Laga AC, Shivdasani P, Chin G, Luke JJ, Ott PA, Hodi FS, 2015 Targeted next-generation sequencing reveals high frequency of mutations in epigenetic regulators across treatment-naive patient melanomas. Clin Epigenetics 7: 59. 26221190 10.1186/s13148-015-0091-3 PMC4517542

[TORDELLAGAD286112C31] Nardella C, Clohessy JG, Alimonti A, Pandolfi PP. 2011 Pro-senescence therapy for cancer treatment. Nat Rev Cancer 11: 503–511. 21701512 10.1038/nrc3057

[TORDELLAGAD286112C32] Narita M, Lowe SW. 2005 Senescence comes of age. Nat Med 11: 920–922. 16145569 10.1038/nm0905-920

[TORDELLAGAD286112C33] Oruetxebarria I, Venturini F, Kekarainen T, Houweling A, Zuijderduijn LM, Mohd-Sarip A, Vries RG, Hoeben RC, Verrijzer CP. 2004 P16INK4a is required for hSNF5 chromatin remodeler-induced cellular senescence in malignant rhabdoid tumor cells. J Biol Chem 279: 3807–3816. 14604992 10.1074/jbc.M309333200

[TORDELLAGAD286112C34] Plass C, Pfister SM, Lindroth AM, Bogatyrova O, Claus R, Lichter P. 2013 Mutations in regulators of the epigenome and their connections to global chromatin patterns in cancer. Nat Rev Genet 14: 765–780. 24105274 10.1038/nrg3554

[TORDELLAGAD286112C35] Robson SC, Sevigny J, Zimmermann H. 2006 The E-NTPDase family of ectonucleotidases: structure function relationships and pathophysiological significance. Purinergic Signal 2: 409–430. 18404480 10.1007/s11302-006-9003-5 PMC2254478

[TORDELLAGAD286112C36] Samartzis EP, Gutsche K, Dedes KJ, Fink D, Stucki M, Imesch P. 2014 Loss of ARID1A expression sensitizes cancer cells to PI3K- and AKT-inhibition. Oncotarget 5: 5295–5303. 24979463 10.18632/oncotarget.2092 PMC4170604

[TORDELLAGAD286112C37] Sausen M, Leary RJ, Jones S, Wu J, Reynolds CP, Liu X, Blackford A, Parmigiani G, Diaz LA Jr, Papadopoulos N, 2013 Integrated genomic analyses identify ARID1A and ARID1B alterations in the childhood cancer neuroblastoma. Nat Genet 45: 12–17. 23202128 10.1038/ng.2493 PMC3557959

[TORDELLAGAD286112C38] Shain AH, Pollack JR. 2013 The spectrum of SWI/SNF mutations, ubiquitous in human cancers. PLoS One 8: e55119. 23355908 10.1371/journal.pone.0055119 PMC3552954

[TORDELLAGAD286112C39] Shen J, Peng Y, Wei L, Zhang W, Yang L, Lan L, Kapoor P, Ju Z, Mo Q, Shih Ie M, 2015 ARID1A deficiency impairs the DNA damage checkpoint and sensitizes cells to PARP inhibitors. Cancer Discov 5: 752–767. 26069190 10.1158/2159-8290.CD-14-0849 PMC4497871

[TORDELLAGAD286112C40] Shi JD, Kukar T, Wang CY, Li QZ, Cruz PE, Davoodi-Semiromi A, Yang P, Gu Y, Lian W, Wu DH, 2001 Molecular cloning and characterization of a novel mammalian endo-apyrase (LALP1). J Biol Chem 276: 17474–17478. 11278936 10.1074/jbc.M011569200

[TORDELLAGAD286112C41] Trouche D, Le Chalony C, Muchardt C, Yaniv M, Kouzarides T. 1997 RB and hbrm cooperate to repress the activation functions of E2F1. Proc Natl Acad Sci 94: 11268–11273. 9326598 10.1073/pnas.94.21.11268 PMC23436

[TORDELLAGAD286112C42] Wang J, Lohman GJ, Stubbe J. 2009 Mechanism of inactivation of human ribonucleotide reductase with p53R2 by gemcitabine 5′-diphosphate. Biochemistry 48: 11612–11621. 19899807 10.1021/bi901588z PMC2917093

[TORDELLAGAD286112C43] Watson IR, Takahashi K, Futreal PA, Chin L. 2013 Emerging patterns of somatic mutations in cancer. Nat Rev Genet 14: 703–718. 24022702 10.1038/nrg3539 PMC4014352

[TORDELLAGAD286112C44] Wilson BG, Roberts CW. 2011 SWI/SNF nucleosome remodellers and cancer. Nat Rev Cancer 11: 481–492. 21654818 10.1038/nrc3068

[TORDELLAGAD286112C45] Zender L, Xue W, Zuber J, Semighini CP, Krasnitz A, Ma B, Zender P, Kubicka S, Luk JM, Schirmacher P, 2008 An oncogenomics-based in vivo RNAi screen identifies tumor suppressors in liver cancer. Cell 135: 852–864. 19012953 10.1016/j.cell.2008.09.061 PMC2990916

