# Supplementary material for: SWI/SNF regulates a transcriptional program that induces senescence to prevent liver cancer
Source: Genes Dev. 2016 Oct 1;30(19):2187–98. doi: 10.1101/gad.286112.116 (PMC5088567; doi:10.1101/gad.286112.116)
Supplement: Supplemental Material [file supp_30_19_2187__index.html]

SWI/SNF regulates a transcriptional program that induces senescence to prevent liver cancer — Supplemental Material 

# SWI/SNF regulates a transcriptional program that induces senescence to prevent liver cancer

## Supplemental Material

- Supplemental\_Material.pdf
