# Supplementary material for: SWI/SNF regulates a transcriptional program that induces senescence to prevent liver cancer
Source: Genes Dev. 2016 Oct 1;30(19):2187–98. doi: 10.1101/gad.286112.116 (PMC5088567; doi:10.1101/gad.286112.116)
Supplement: Supplemental Material [file supp_gad.286112.116_Supplemental_Material.pdf]

SUPPLEMENTAL INFORMATION for

SWI/SNF regulates a transcriptional programme that induces senescence to prevent liver cancer

Luca Tordella, Sadaf Khan, Anja Hohmeyer, Ana Banito, Sabrina Klotz, Selina Raguz, Nadine Martin, Gopuraja Dhamarlingam, Thomas Carroll, José Mario González Meljem, Sumit Deswal, Juan Pedro Martínez-Barbera, Ramón García-Escudero, Johannes Zuber, Lars Zender and Jesús Gil

Including:

- **Supplemental Figures S1 to S9 and legends**
- **Supplemental Tables S1 to S4**
- **Supplemental Materials and Methods**
- **Supplemental References**

SUPPLEMENTAL FIGURE LEGENDS

Figure S1. *ARID1B* is a novel regulator of senescence.

(A) Timeline of the screen. The screen was performed in triplicate and samples were collected at indicated time points to analyze shRNA enrichment. (B) shRNA enrichment analysis. The x-axis shows the $\log_2$ median of the shRNAs fold change (FC) at the four different time points analysed for the three replicates. The y-axis shows the $-\log_{10}$ of the Fisher's combined p value of each shRNA. Significantly enriched shRNAs (at $p < 0.001$) are marked in blue. Among those, shRNAs targeting ARID1B are marked in red. (C) qRT-PCR analysis of *ARID1B* and *INK4a* mRNA levels in MEFs transduced with an empty vector or shRNAs targeting $p16^{INK4a}$ (shp16) or Arid1b (shArid1b.1, shArid1b.2). (D) In the same conditions, knockdown of Arid1b was also assessed by western blot using an anti-ARID1B antibody. An anti-α-Tubulin antibody was used as loading control. (E) MEFs were infected with the indicated constructs and subjected to colony formation assay (left). mRNA quantification of the indicated genes by qRT-PCR is shown on the right (vec., vector). Graphs represent mean ± SD from *n=3* (C) and *n=2* (E, right). $^{***}p < 0.001$ by two-tailed Student *t*-test.

Figure S2. *ARID1B* depletion blunts oncogene-induced-senescence (OIS) *in vivo.*

(A) Representative IF images of liver sections from mice injected with the indicated constructs and co-stained with antibodies against Nras and ARID1B are shown. DAPI was used to visualize the nuclei. Higher magnification views of the boxed regions are shown. Arrows indicate ARID1B nuclear staining within Ras^{G12V}-expressing hepatocytes. (B) qRT-PCR mRNA analysis of *Arid1b* in livers of mice injected with the indicated constructs and sacrificed after 9 days. *n=4* mice per condition. The graph represents mean ± SD. (C) Representative IF images of liver sections co-stained with antibodies against Nras and 53BP1 are shown. DAPI was used to visualize the nuclei. Arrows indicate 53BP1 nuclear staining within Ras^{G12V}-expressing hepatocytes. (D) Representative IF images of liver

sections from mice injected with the indicated constructs and co-stained with antibodies against Nras and Ki67 are shown. DAPI was used to visualize the nuclei. Inset, higher magnification views. Scale bars, 10 μm.

Figure S3. ARID1B regulates OIS in human cells.

(A) qRT-PCR analysis of *TP53* and *ARID1B* mRNA levels in IMR90 cells transduced with an empty vector (vec) or shRNAs targeting p53 (shp53) or ARID1B (shA.2, shA.3). (B) In the same conditions, knockdown of ARID1B was also assessed by western blot using an anti-ARID1B antibody. An anti-α-Tubulin antibody was used as loading control. (C) qRT-PCR analysis of *CDKN1A* (left) and *INK4A* (right) mRNA levels in IMR90 ER:RAS cells transduced with an empty vector (vec) or shRNAs targeting p53 (shp53), p16 (shp16) or ARID1B (shA.2, shA.3) and induced to undergo senescence by 4OHT treatment. Graphs represent mean ± SD from *n=2*.

Figure S4. Ectopic expression of *ARID1B* induces senescence.

(A) IMR90 cells were transduced with either an empty vector, *ARID1B* cDNA or Ras^{G12V} cDNA and then immunostained using an anti-ARID1B antibody. Representative pictures of the IF staining (left) and quantification by HCA (right) are shown. DAPI was used to visualize the nuclei. Scale bar 30 μm. (B) GSEA of OIS, SAPS and cell-cycle gene (RB1 targets) signatures in the gene expression profile of IMR90 transduced with *ARID1B* cDNA versus empty vector. NES, normalized enriched score; FDR, false discovery rate. (C) qRT-PCR mRNA analysis of *TP53*, *CDKN1A* and *INK4A* in IMR90 ER:RAS transduced with an empty vector (vec) or shRNAs targeting p53, $p21^{CIP1}$ or $p16^{INK4a}$. Graphs represent mean ± SD from *n=4* (A) and *n=2* (C). $^{***}p < 0.001$ by two-tailed Student *t*-test.

Figure S5. A focused shRNA screen identifies novel ARID1B effectors controlling senescence.

(A) Genes selected to generate the focused screen shRNA library. The x axis shows the $\log_2$ FC between IMR90 ER:RAS + 4OHT and – 4OHT (Ras induction), while the y axis the $\log_2$ FC between IMR90 ER:RAS vector and shARID1B, both 4OHT treated (ARID1B dependency). Genes targeted in the shRNA library are marked in colours other than grey [(x > 1; y < 0) U (x > 0; y < 0.4)]. Blue; genes with a ratio value between 2 and 0.5 (induced by RAS, dependent on ARID1B). Orange; genes with a ratio value > 2 (induced by Ras, independently of ARID1B). Yellow; genes with a ratio value < 0.5 (induced by ARID1B only). (B) Timeline of the screen is shown. The screen was performed in duplicate. (C) Enrichment for Ras-induced ARID1B-dependent genes in the screen. Comparison between the distribution of genes belonging to the three groups in the library (left, 43%, 44% and 13%, respectively) and in the top hits of the screen (right, Fisher's combined p value < 0.001, 88% of genes induced by Ras and dependent on ARID1B; 12% of genes induced by Ras independently of ARID1B). (D) IPA upstream regulator analysis of the screen top hits (p < 0.05) reveals activation (z-score) of SMARCA4, TP53 and ROS pathways. (E) shRNA enrichment analysis. The x axis shows the $\log_2$ median FC of the multiple shRNAs per gene in both replicates. The y axis shows the $-\log_{10}$ of the Fisher's combined p value calculated for each gene. Significantly enriched shRNAs (at p < 0.001) are marked in blue. Among those, genes selected for further analysis are marked in red, with *CDKN1A* used as internal control. (F) qRT-PCR analysis of the expression of the indicated genes in IMR90 transduced with either an empty vector or cDNA of *ARID1A*, *ARID1B* or *BRG1*. (G) ENCODE database analysis of the co-localization of marks for transcription initiation (H3K4me3, H3K27Ac and Dnase hypersensitive sites) with SMARCC1 (coding for SWI/SNF core subunit BAF155) ChIP-seq at the promoter region of the indicated genes in HeLa cells. (H) qRT-PCR analysis of the expression of the indicated genes in IMR90 ER:RAS transduced with either an empty vector or shRNAs targeting ARID1B (shA.3), ENTPD7 (shE.2), SLC31A2 (shS.2) or NDST2 (shN.2), ± 4OHT. Graphs represent mean ± SD from *n=3* (F and H). ***p < 0.001; **p < 0.005; *p < 0.05 by two-tailed Student *t*-test.

Figure S6. Individual knockdown of ENTPD7, SLC31A2 and NDST2 allows bypass of senescence.

(A) qRT-PCR analysis of respectively *ENTPD7*, *SLC31A2* and *NDST2* mRNA levels in IMR90 transduced with two shRNAs targeting ENTPD7, SLC31A2 or NDST2. (B) RAS/ERK pathway activation was assessed by western blot using anti-phospho p42/44, total p42/44 and Hras antibodies in IMR90 co-transduced with Ras^{G12V} cDNA (or empty vector) and shRNAs targeting ENTPD7 (shE.2, shE.3), SLC31A2 (shS.1, shS.2), NDST2 (shN.1, shN.2) or p53. An anti-α-Tubulin antibody was used as loading control. (C and D) BrdU incorporation HCA analysis (C) and colony formation assay (D) in IMR90 co-transduced with *ARID1B* cDNA (or empty vector) and shRNAs targeting ENTPD7 (shE.2, shE.3), SLC31A2 (shS.1, shS.2), NDST2 (shN.1, shN.2) or p53. Graphs represent mean ± SD from *n=2* (A) and *n=3* (C). $^{***}p < 0.001$; $^{**}p < 0.005$; $^{*}p < 0.05$ by two-tailed Student *t*-test.

Figure S7. *ARID1B* controls senescence by regulating multiple targets.

(A) qRT-PCR mRNA analysis of *Entpd7*, *Slc31a2* and *Ndst2* in livers of mice injected with the indicated constructs and sacrificed after 6 days. *n=3* mice per condition. (B) Copy number loss gene rank in TGCA liver shows decreased expression of ARID1B, SLC31A2 and ENTPD7 in hepatocellular carcinoma compared with normal liver samples (source: Oncomine). (C) qRT-PCR mRNA analysis of *Arid1b, Entpd7* and *Slc31a2* in livers of mice injected with the indicated constructs and sacrificed after 6 days. *n=4* mice per condition, except for control mice *n=6*. (D) qRT-PCR mRNA analysis of *Entpd7* and *Slc31a2* in livers of mice injected with the indicated constructs and sacrificed after 9 days. *n=3* mice per condition. Graphs represent mean ± SD. $^{**}p < 0.005$; $^{*}p < 0.05$; n.s. non-significant by two-tailed Student *t*-test.

Figure S8. *ENTPD7* is a novel *ARID1B* target-gene controlling senescence.

(A) Scatter plot of ARID1B and ENTPD7 expression, showing highly significant co-expression in the TCGA liver HCC dataset, as tested using Pearson correlation (r=0.39).

Green line denotes linear regression. (B) Pearson correlation values between ENTPD7 and ARID1B expression in primary tumours from different cancer cohorts from the TCGA Pan-cancer (PANCAN) dataset. Only significant correlations are shown (p value < 0.05) across different tumour types. HCC is highlighted in red. (C) The pyrimidine ribonucleotides biosynthesis pathway was identified in the gene expression profile of IMR90 transduced with *ARID1B* cDNA versus empty vector by IPA canonical pathway analysis. (D) Representative images of co-IF in IMR90 cells transduced with an HA-tagged *ENTPD7* cDNA, using an HA-tag antibody in combination with antibodies marking different intracellular organelles, as indicated in the figure. DAPI was used to visualize the nuclei. Scale bar, 10 µm. (E) IMR90 cells were transduced either with an empty vector, *ARID1B* cDNA or *ENTPD7* cDNA and then indicated intracellular dNTP levels were measured. (F) IMR90 ER:RAS cells were transduced with either an empty vector, an ARID1B shRNA or an ENTPD7 shRNA and induced to undergo senescence by 4OHT treatment. Indicated intracellular dNTP levels were measured 5 days after 4OHT. Graphs in E and F represent mean ± SD from n=3. ***p < 0.001; **p < 0.005; *p < 0.05; n.s. non-significant by two-tailed Student *t*-test.

Figure S9. Nucleotide metabolism is a liability of ARID1B-deficient cells.

(A) Scheme indicating the role of ARID1B signalling in senescence and a suggested therapeutic approach for ARID1B-mutated tumours. ARID1B regulates a complex senescence response upon oncogene activation, leading to induction of cell cycle regulators p16 and p12, but also of a set of newly discovered targets (ENTPD7, SLC31A2 and NDST2), which contribute to p53 activation via generation of DNA damage and ROS. The blue arrows indicate a pro-senescence therapeutic approach for ARID1B mutated tumours using drugs (DON, Gemcitabine) which mimic the function of ARID1B-downstream mediators, such as ENTPD7. (DDR, DNA-damage response; ROS, reactive oxygen species). (B) Cell growth assay (crystal violet) and quantification of IMR90 ER:RAS cells co-transduced with an empty vector or *ENTPD7* cDNA and shRNAs targeting ARID1B, p53 or

control (+ 4OHT treatment). (C) Cell growth assay (crystal violet) and quantification of stable ARID1B-knockdown, p53-knockdown or control IMR90 ER:RAS cells treated with 4OHT, followed by Gemcitabine (5 μM) or DMSO. The graphs represent mean ± SD from *n*=2. $^{***}p < 0.001$ by two-tailed Student *t*-test.

Table S1. shRNA target sequences

Construct	Target sequence
MLP shp16/Arf (m)	CCGCTGGGTGCTCTTTGTGT
pRS shARID1B-2 (h)	AAGCAAATTGACTTTAAAGAA
pRS shARID1B-3 (h)	ACCATGAAGACTTGAACTTAA
pRS shp16 (h)	GAGGAGGTGCGGGCGCTGC
pRS shp53 (h)	GTAGATTACCACTGGAGTC
pRS shp21 (h)	TCCCACAATGCTGAATATACA
pRRL shARID1B.1 (m)	AAAGGCGAAAGATTACCTCAAA
pRRL shARID1B.2 (m)	CTACGAAGTGATGGTAGATTTA
pRRL shp53 (m)	TAACTGCAAGAACATTTCT
pRRL shENTPD7.2 (h)	CCACGAGATAGGCAATACGAAA
pRRL shENTPD7.3 (h)	GTCAGTCTAAGTTACAGACAAA
pRRL shSLC31A2.1 (h)	ATGGGCAGGTACTGACTGAAGA
pRRL shSLC31A2.2 (h)	GTCTATGGATCATGTTGACAAA
pRRL shNDST2.1 (h)	GCAGCAGTGTATCTATTCTATA
pRRL shNDST2.2 (h)	CCGCAGCAGTGTATCTATTCTA
CaNIGmirE-shRenilla	CAGGAATTATAATGCTTATCTA
CaNIGmirE-shARID1B.1 (m)	AAAGGCGAAAGATTACCTCAAA
CaNIGmirE-shARID1B.2 (m)	CTACGAAGTGATGGTAGATTTA
CaNIGmirE-shENTPD7.1 (m)	ATAGGTTAAGAACTCTGTTATA
CaNIGmirE-shENTPD7.2 (m)	TGAGGATGAGTGTGTTGACTTA
CaNIGmirE-shSLC31A2.1 (m)	TCCAGATCAACTTCAGACAATA
CaNIGmirE-shSLC31A2.2 (m)	TAAGCAAGTAGCTGTGACCAAA

*h, human; m, mouse.

Table S2. cDNA constructs.

Vector	cDNA
pBabe	Empty vector
pBabe HA-tag	Empty vector
pBabe	ARID1B
pBabe	BRG1
pBabe	RAS^{G12V}
pBabe	mENTPD7
pBabe HA-tag	mENTPD7
pLenti	ARID1A
pRS	Empty vector
pMSCV	RAS^{G12V}
pMSCV	Empty vector
pRRL	Empty vector
CaNIGmirE-5’	$RAS^{G12V/D38A}$
CaNIGmirE-5’	RAS^{G12V}

Table S3. Primers used for RT-qPCR.

Name	Sequence (5'-3')
hARID1B F	CTGTCGCCTCAGAGAC
hARID1B R	GTTCGATAAAAGCGCCAT
mARID1B F	TCAGCAGTCAGTGCATCCG
mARID1B R	CCAGACCTCGATTGGTTGCTT
hCDKN1a F	CCTGTCACTGTCTTGTACCCT
hCDKN1a R	GCGTTTGGAGTGGTAGAAATCT
hENTPD7 F	ACAGATGAGAGACCGCAACAG
hENTPD7 R	GCAGAGGACGAAGGTAATCACTG
mENTPD7 F	CACTGGGCCACTTCGTTACC
mENTPD7 R	CCACCACGAGTCCATAATTCAGA
hINK4a F	CGGTCGGAGGCCGATCCAG
hINK4a R	GCGCCGTGGAGCAGCAGCAGCT
mINK4a F	CCCAACGCCCCGAACT
mINK4a R	GCAGAAGAGCTGCTACGTGAA
hRPS14 F	GACCAAGACCCCTGGACCT
hRPS14 R	CCCCTTTTCTTCGAGTGCTA
mRPS14 F	GACCAAGACCCCTGGACCT
mRPS14 R	CCCCTTTTCTTCGAGTGCTA
hSLC31A2 F	GCAGACCATCGCAGAGACAG
hSLC31A2 R	GCCAAAGTGACACAAATACCAC
mSLC31A2 F	CACAGCCCTACAGGCATGG
mSLC31A2 R	CCAGAGTCTTGTGGAGCAATTTG
hTP53 F	CCGCAGTCAGATCCTAGCG
hTP53R	AATCATCCATTGCTTGGGACG
mTP53 F	CACGTACTCTCCTCCCCTCAAT
mTP53 R	AACTGCACAGGGCACGTCTT

*F, forward; R, reverse; h, human; m, mouse.

Table S4. Antibodies.

Antibody	Application
ARID1B (Abgent - AT1189a)	IF, IHC/IF, WB
BrdU (BD Pharmigen - 555627)	IF
p16^{INK4a} (Santa Cruz - sc-56330)	IF
p16^{INK4a} (Santa Cruz - sc-1207)	IF, IHC/IF
p21^{Cip1} (Sigma - P1484)	IF
p21^{Cip1} (Santa Cruz - sc-471)	IF, IHC/IF
NRas (Santa Cruz - sc-31)	IHC, IHC/IF
NRas (Abcam - ab77392)	IHC, IHC/IF
8-Oxoguanine (Millipore - MAB3560)	IF
p53 (Santa Cruz - sc-126)	IF
53BP1 (Novus – NB100-304)	IF, IHC/IF
γH2AX (Millipore – 05-636)	IF
Ki67 (Abcam - ab77392)	IHC/IF
PDI (Abcam - ab2792)	IF
Tom20 (Santa Cruz - sc-11415)	IF
GM-130 (BD Biosciences - 610822)	IF
HA-tag (Sigma - 3F10)	IF
Hras (Santa Cruz - sc-520)	WB
p42/44 (Cell Signaling - 4695)	WB
Phospho-p42/44 (Cell Signaling - 9101)	WB
α-Tubulin (Santa Cruz – sc-8035)	WB

*IF, immunofluorescence; IHC, immunohistochemistry; WB, Western blot

SUPPLEMENTAL MATERIALS AND METHODS

Cell culture, generation of primary MEFs, retroviral and lentiviral infection

All cells lines were maintained in Dulbecco's modified Eagle's medium (Invitrogen) supplemented with 10% foetal bovine serum (Sigma), 1% antibiotic-antimycotic solution (Invitrogen). Cell number and viability measurements were determined using the Guava Viacount reagent (Millipore) and the Guava Cytometer (Millipore). IMR90 ER:RAS cells were generated and treated with 100 nM 4OHT to induce senescence as previously described (Barradas et al. 2009). For the experiments with 6-Diazo-5-oxo-L-norleucine (DON, Sigma) and Gemcitabine (Sigma), $4x10^4$ IMR90 ER:RAS were plated into 6-well plates and treated with 4OHT the following day. 1-5 µM of either of the drugs (or DMSO) was added to the medium after two days and again after four days. Two days later cells were fixed and stained with Crystal violet. Quantification was performed by dissolution in 10% acetic acid and absorbance measurement. MEFs were prepared from 13.5-day-old embryos of CD1 mice. The head and viscera were removed and the remaining tissue was minced. A single cell suspension was obtained by trypsinisation (0.1% (v/v)) (Gibco, UK) at 37°C for 15 min. Cells were cultured until they became confluent and were then frozen in foetal bovine serum (FBS; Sigma) containing 10% dimethyl sulfoxide (DMSO; Sigma). Retroviral and lentiviral infection were performed as previously described (Banito et al. 2009; Barradas et al. 2009).

Plasmids

pLNC-ER:RAS has been described previously (Acosta et al. 2008). For *de novo* generation of miR-E-based shRNAs, 97-mer oligonucleotides coding for the respective shRNAs (Fellmann et al. 2013) were PCR amplified using the primers miRE-Xho-fw and miRE-EcoOligo-rev and cloned into the pRRL lentiviral backbone. Sequences used for RNA interference are listed in Table S1. For the *in vivo* experiments, shRNAs were excised from

their pRRL backbone and subcloned into the transposon-based Nras expression plasmid CaNIGmirE-5'. The pBabe ENTPD7 plasmid was obtained from GenScript. ENTPD7 cDNA was subsequently sub-cloned (BamHI, SalI) into an N-terminal HA-tagged pBabe empty vector. All expression plasmids used in the study are listed in Table S2.

shRNA libraries

To screen genes deleted in HCC with a role in regulating senescence, we took advantage of a previously described shRNA library. This shRNA library consists of 631 miR30-based shRNAs, targeting 301 mouse orthologs of genes deleted in HCC cloned in a murine stem cell virus (MSCV)-SV40-GFP vector (Zender et al. 2008).

To identify ARID1B-targets controlling senescence, we designed a library consisting of 1,504 miRE-based shRNAs, targeting 255 human genes (most genes were targeted by 6 shRNAs each). To select the targeting sequences, we used sensor-based shRNA predictions (Fellmann et al. 2011; Fellmann et al. 2013). Oligonucleotides (136 nt-long oligonucleotides containing 97 nt coding for the respective shRNAs, an EcoRI cloning site and a 20 nt adaptor site) were synthesized on an oligonucleotide array (MYcroarray) (Cleary et al. 2004) and pooled for cloning (Cleary et al. 2004; Fellmann et al. 2011). The pool of oligonucleotides was PCR-amplified and cloned through XhoI/EcoRI sites into the pRRL-SFFV-GFP-miRE-PGK-Puro vector (Fellmann et al. 2013).

Preparation of libraries for determining shRNA enrichment in the screens

DNA was extracted from infected pools using DNeasy Blood and Tissue Kit (Qiagen) as described by the manufacturer. 1μg of extracted DNA was used for PCR with forward and reverse primers ligated to Solexa adaptors. The forward primer used for the PCR also contains a 3-nucleotide barcode, which is unique and corresponds to the time point at which the infected pool was collected. PCR-products were extracted from gels; each barcoded sample was extracted as an individual product using a MinElute Gel Extraction Kit (Qiagen) and quantified using the Qubit 2.0 Fluorometer. The individual barcoded PCR-products

were then pooled in equal quantities to form a Solexa sequencing library. Quality control of the Solexa library involved assessment of the size of the PCR product (Agilent High Sensitivity DNA Kit, Agilent 2100 Bioanalyser) and the amount of amplifiable DNA (qPCR). 6pM of the Solexa library was used to create clusters and sequenced on a Genome Analyser IIx.

Statistical analysis of the shRNA screens

Fasta files produced from the sequencing runs were processed and sequences were demultiplexed with CASAVA 1.7. The reverse complement of each read was aligned to the custom shRNA libraries using ELAND. Candidates were ranked using Fisher's combined p-value and edgeR. The Fisher's combined test allows p-values across independent data sets to be combined, bearing upon the same overall hypothesis (Fisher 1925).

Microarray and RNA-Sequencing

For microarray experiments, cRNA was hybridized to Human Gene 1.0 ST arrays (Affymetrix) following manufacturer's instructions. Three biological replicates were performed for each condition. Microarray data processing and analysis was carried out at EMBL-GeneCore (Heidelberg, Germany). Microarray data was normalized using Robust Multichip Average (RMA) method available in "oligo" Bioconductor package and significant differentially expressed probesets were identified using Limma (Ritchie et al. 2015) with Benjamini-Hochberg corrected p-value < 0.05.

RNA-Seq libraries were prepared from 500 ng of total RNA using the Illumina Truseq mRNA stranded library prep kit (Illumina Inc. San Diego, USA) according to the manufacturer's protocol. Library quality was checked on a Bioanalyser HS DNA chip and concentrations were estimated by Qubit measurement. Libraries were pooled in equimolar quantities and sequenced on a Hiseq2500 using paired end 100 bp reads. At least 40 million reads passing filter were achieved per sample. Sequencing reads from the RNA-Seq experiments were aligned to hg19 genome using Tophat v2.0.11 (Trapnell et al. 2009) using parameters

"--library-type fr-firststrand --b2-very-sensitive --b2-L 25" and using known transcripts annotation from ensembl gene v72. Number of reads counts on exons were summarised using HTSeq v0.5.3p9 with "--stranded=reverse" option and differentially expressed genes were identified using DESeq2 (Love et al. 2014). Genes were ranked by fold change and Gene Set Enrichment Analysis was performed using GSEA v2.07 (Broad Institute) pre-ranked module. The activity of signalling pathways was identified using Ingenuity Pathways Analysis software (IPA, QIAGEN). Data was analysed using IPA's Upstream Regulator analysis ($P < 0.05$) to predict activation or inhibition (z-score) of transcriptional regulators. This analysis is based on prior knowledge of expected effects between transcriptional regulators and their targets stored in the Ingenuity® Knowledge Base.

Immunoblot

Protein extracts from cell lines were processed and analysed as previously described (Barradas et al. 2009). The antibodies used are listed in Table S4.

ARID1B and ENTPD7 expression correlation analysis

TCGA Pan-cancer (PANCAN) expression values were selected from the "gene expression" dataset, measured using the IlluminaHiSeq technology. Correlation was calculated per cohort using Pearson test.

SWI/SNF mutation and ENTPD7 expression analyses in TCGA liver hepatocellular carcinoma (LIHC)

TCGA data was downloaded from UCSC Xena tool (http://xena.ucsc.edu/). Primary tumour samples having mutation in any of the SWI/SNF complex gene members (ACTL6A, ACTL6B, ARID1A, ARID1B, ARID2, BRD7, DPF1, DPF2, DPF3, PBRM1, PHF10, SMARCA2, SMARCA4, SMARCB1, SMARCC1, SMARCC2, SMARCD1, SMARCD2, SMARCD3, and SMARCE1) were selected from the "somatic mutation SNPs and small INDELs (bcm automated)" dataset. Sequencing data were generated on an IlluminaGA

system. The calls are generated at Baylor College of Medicine Human Genome Sequencing Center using the Baylor pipeline method. When variant allele frequency information is available, only calls with VAF >10% are kept. ENTPD7 expression values were selected from the “gene expression RNAseq (IlluminaHiSeq percentile)” dataset. ARID1B wt, n=184; ARID1B mut, n=11; SWI/SNF wt, n=132; SWI/SNF mut, n=63. Differential expression analysis was done using Student’s *t*-test.

Immunofluorescence for high content analysis

IF was performed as previously described (Banito et al. 2009) using the antibodies listed in Table S4. Image acquisition was performed using an automated high throughput microscope (IN Cell Analyzer 2000, GE Healthcare). A minimum of 1,000 cells was acquired for each sample per duplicate. High content analysis (HCA) was performed using the IN Cell Investigator software (v 3.2; GE Healthcare), as described elsewhere (Banito et al. 2009; Barradas et al. 2009; Bishop et al. 2010; Acosta et al. 2013). Briefly, DAPI staining of the nuclei was used to identify cells. The nuclei were segmented using top-hat segmentation, specifying a minimum nucleus area of 100 μm^2. To define the cell area, a collar segmentation approach was used with a border of 1 μm around DAPI staining or alternatively, multiscale top-hat was used to detect cytoplasmic intensity for a given staining. Each cell was assigned a nuclear intensity value (and cell intensity value when applicable) for the specific protein being studied. A histogram of the intensity values of all the cells in a sample was produced and this was used to set a threshold filter to determine positive and negative expressing cells. Alternatively, normalized intensity values for a given staining were obtained by subtracting the intensity of the secondary antibody alone (background intensity) to raw intensity values. Normalized intensity values were used to calculate fold changes among different conditions. The antibodies used for the analysis were validated with appropriate controls (shRNAs and/or overexpression vectors) to assess their specificity. Statistical analysis of IF data was conducted using GraphPad software Prism® (version 6.0b). Unpaired Student’s *t*-test was used to calculate p-values.

Staining of tissue sections

Rehydrated paraffin-embedded liver sections (4μm) were subjected to antigen retrieval treatment for 20 minutes in a steamer in 10 mM sodium citrate buffer, pH 6. Then, sections were incubated in 3% (vol/vol) hydrogen peroxide, washed in PBS solution, blocked with 5% (vol/vol) goat serum in PBS solution for 1 h at room temperature (RT) and incubated overnight (O/N) at 4 °C with the primary antibody diluted in blocking solution. Subsequently, sections were incubated with biotinylated or Alexa Fluor (1:400; Molecular Probes) secondary antibody for 30 min at RT and then washed in PBS solution. Sections incubated with fluorescent secondary antibodies were mounted and analysed by confocal microscopy (Zeiss) or by high throughput microscope (IN Cell Analyzer 2000, GE Healthcare), whereas sections incubated with biotinylated secondary antibodies were first treated with the peroxide substrate solution diaminobenzidine (Vector), followed by dehydration, mounting, and analysis by phase-contrast microscopy. Fluorescence intensities were quantified using ImageJ Software (NIH), while the number of positive cells was quantified using the IN Cell Investigator software. Frozen sections were used for SA-β-Gal staining. The staining was performed as in cultured cells (after thawing the sections). After the staining, the slides were counterstained with eosin, dehydrated, mounted and analysed by phase-contrast microscopy. SA-β-Gal tissue staining was quantified using ImageJ Software (NIH) by measuring the percentage of stained area in each section and multiplying it by its mean intensity value.

Measurement of dNTP concentrations in cells

Samples were harvested and dNTP levels were measured as previously described (Wilson et al. 2011).

Accession numbers

Microarray (shARID1B experiment) and RNASeq (ARID1B overexpression experiment) data have been deposited at the Gene Expression Omnibus under the accession numbers GSE75207 and GSE75291 respectively.

SUPPLEMENTAL REFERENCES

Acosta JC, Banito A, Wuestefeld T, Georgilis A, Janich P, Morton JP, Athineos D, Kang TW, Lasitschka F, Andrulis M et al. 2013. A complex secretory program orchestrated by the inflammasome controls paracrine senescence. *Nat Cell Biol* **15**: 978-990.

Acosta JC, O'Loghlen A, Banito A, Guijarro MV, Augert A, Raguz S, Fumagalli M, Da Costa M, Brown C, Popov N et al. 2008. Chemokine signaling via the CXCR2 receptor reinforces senescence. *Cell* **133**: 1006-1018.

Banito A, Rashid ST, Acosta JC, Li S, Pereira CF, Geti I, Pinho S, Silva JC, Azuara V, Walsh M et al. 2009. Senescence impairs successful reprogramming to pluripotent stem cells. *Genes Dev* **23**: 2134-2139.

Barradas M, Anderton E, Acosta JC, Li S, Banito A, Rodriguez-Niedenfuhr M, Maertens G, Banck M, Zhou MM, Walsh MJ et al. 2009. Histone demethylase JMJD3 contributes to epigenetic control of INK4a/ARF by oncogenic RAS. *Genes Dev* **23**: 1177-1182.

Bishop CL, Bergin AM, Fessart D, Borgdorff V, Hatzimasoura E, Garbe JC, Stampfer MR, Koh J, Beach DH. 2010. Primary cilium-dependent and -independent Hedgehog signaling inhibits p16(INK4A). *Mol Cell* **40**: 533-547.

Cleary MA, Kilian K, Wang Y, Bradshaw J, Cavet G, Ge W, Kulkarni A, Paddison PJ, Chang K, Sheth N et al. 2004. Production of complex nucleic acid libraries using highly parallel in situ oligonucleotide synthesis. *Nature methods* **1**: 241-248.

Fellmann C, Hoffmann T, Sridhar V, Hopfgartner B, Muhar M, Roth M, Lai DY, Barbosa IA, Kwon JS, Guan Y et al. 2013. An optimized microRNA backbone for effective single-copy RNAi. *Cell Rep* **5**: 1704-1713.

Fellmann C, Zuber J, McJunkin K, Chang K, Malone CD, Dickins RA, Xu Q, Hengartner MO, Elledge SJ, Hannon GJ et al. 2011. Functional identification of optimized RNAi triggers using a massively parallel sensor assay. *Mol Cell* **41**: 733-746.

Fisher RA. 1925. The Resemblance between Twins, a Statistical Examination of Lauterbach's Measurements. *Genetics* **10**: 569-579.

Love MI, Huber W, Anders S. 2014. Moderated estimation of fold change and dispersion for RNA-seq data with DESeq2. *Genome Biol* **15**: 550.

Ritchie ME, Phipson B, Wu D, Hu Y, Law CW, Shi W, Smyth GK. 2015. limma powers differential expression analyses for RNA-sequencing and microarray studies. *Nucleic Acids Res* **43**: e47.

Trapnell C, Pachter L, Salzberg SL. 2009. TopHat: discovering splice junctions with RNA-Seq. *Bioinformatics* **25**: 1105-1111.

Wilson PM, Labonte MJ, Russell J, Louie S, Ghobrial AA, Ladner RD. 2011. A novel fluorescence-based assay for the rapid detection and quantification of cellular deoxyribonucleoside triphosphates. *Nucleic Acids Res* **39**: e112.

Zender L, Xue W, Zuber J, Semighini CP, Krasnitz A, Ma B, Zender P, Kubicka S, Luk JM, Schirmacher P et al. 2008. An oncogenomics-based in vivo RNAi screen identifies tumor suppressors in liver cancer. *Cell* **135**: 852-864.

Fig. S1

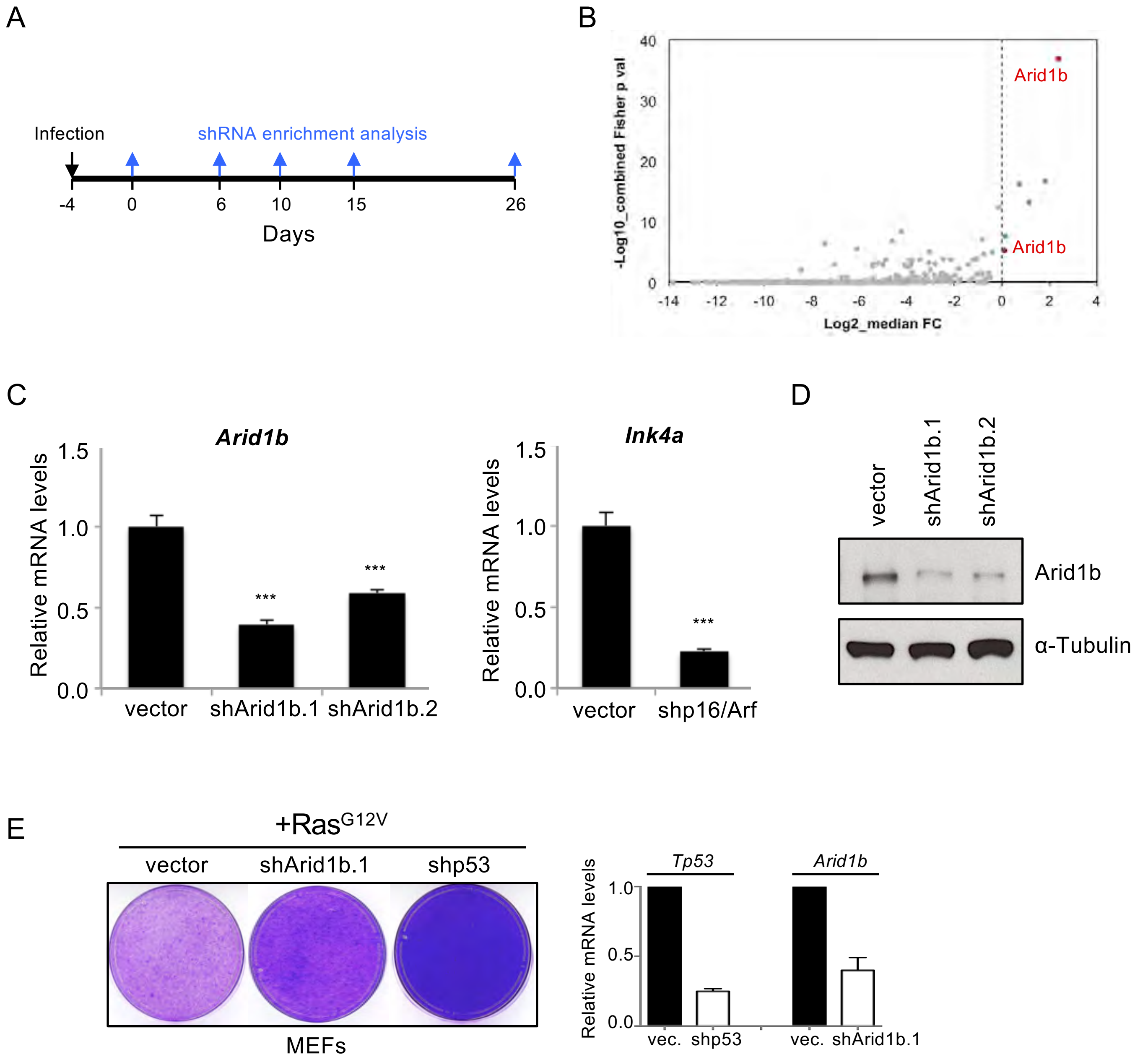
A
Infection
shRNA enrichment analysis
-4
0
6
10
15
26
Days
B
-Log10_combined Fisher p val
Log2_median FC
Arid1b
Arid1b
C
Arid1b
Ink4a
Relative mRNA levels
vector
shArid1b.1
shArid1b.2
shp16/Arf
***
D
vector
shArid1b.1
shArid1b.2
Arid1b
α-Tubulin
E
+RasG12V
vector
shArid1b.1
shp53
MEFs
Tp53
Arid1b
Relative mRNA levels
vec.
shp53
shArid1b.1

Fig. S2

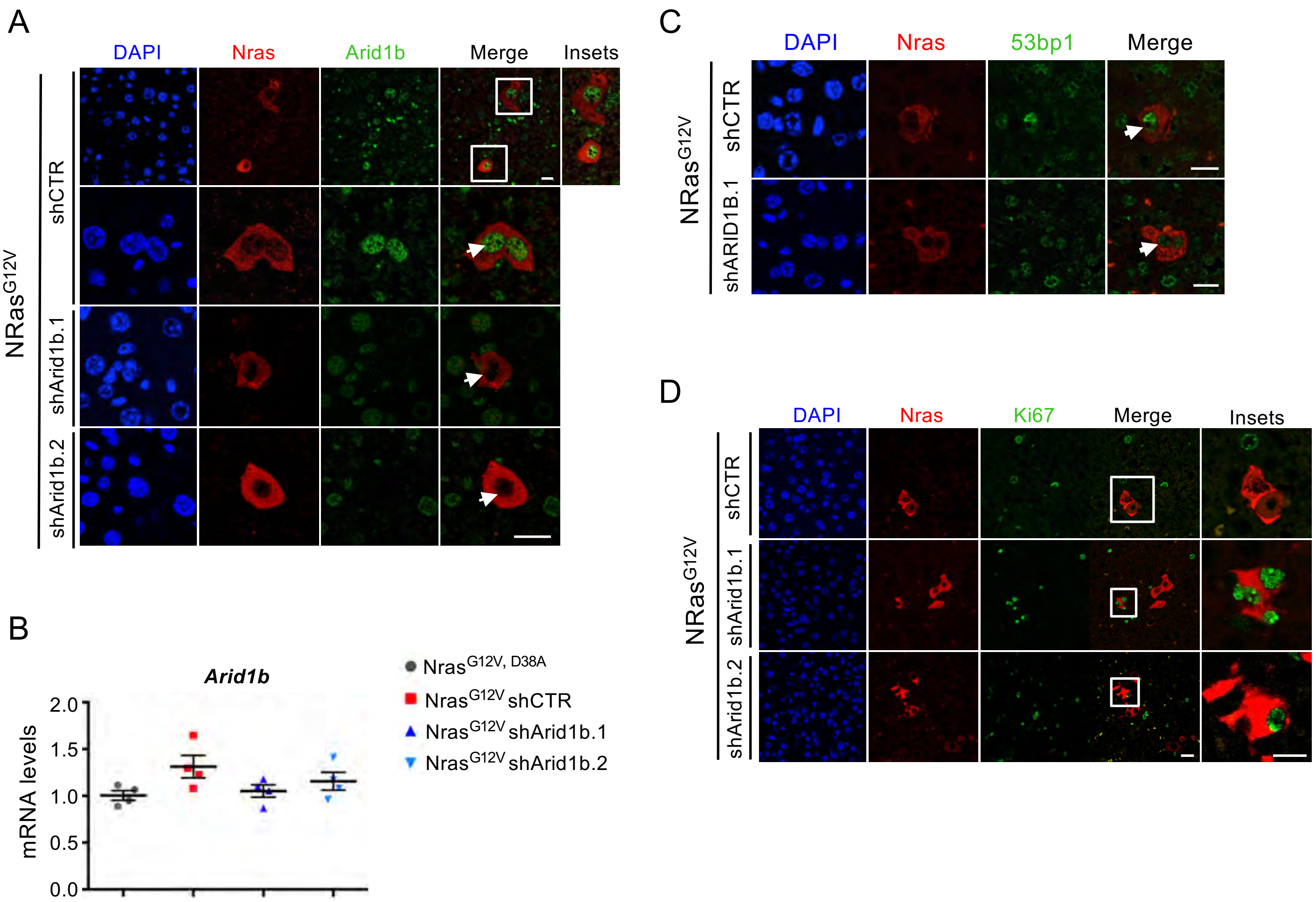

A
DAPI
Nras
Arid1b
Merge
Insets
NRasG12V
shCTR
shArid1b.1
shArid1b.2
B
Arid1b
mRNA levels
2.0
1.5
1.0
0.5
0.0
NrasG12V, D38A
NrasG12V shCTR
NrasG12V shArid1b.1
NrasG12V shArid1b.2
C
DAPI
Nras
53bp1
Merge
NRasG12V
shCTR
shARID1B.1
D
DAPI
Nras
Ki67
Merge
Insets
NRasG12V
shCTR
shArid1b.1
shArid1b.2

Fig. S3

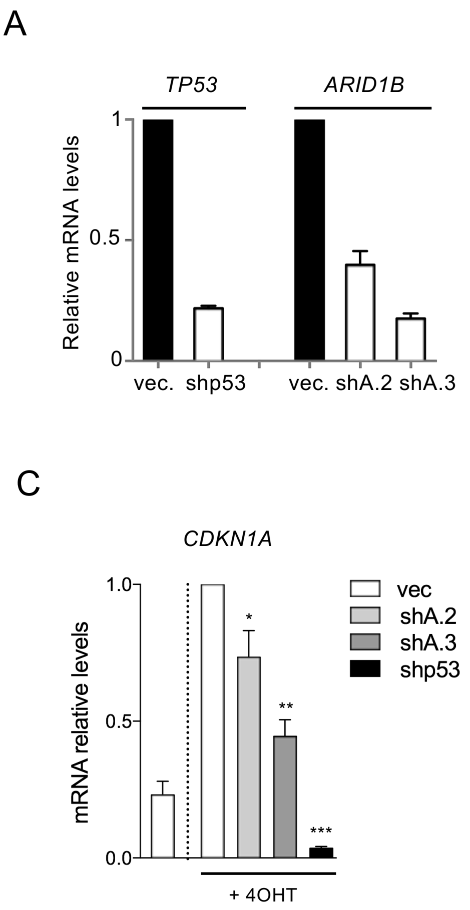
A
TP53
ARID1B
Relative mRNA levels
1
0.5
0
vec.
shp53
vec.
shA.2
shA.3
B
vector
shARID1B.2
shARID1B.3
ARID1B
α–Tubulin
C
CDKN1A
mRNA relative levels
1.0
0.5
0.0
+ 4OHT
vec
shA.2
shA.3
shp53
INK4A
mRNA relative levels
1.0
0.5
0.0
+ 4OHT
vec
shA.2
shA.3
shp16

Fig. S4

A

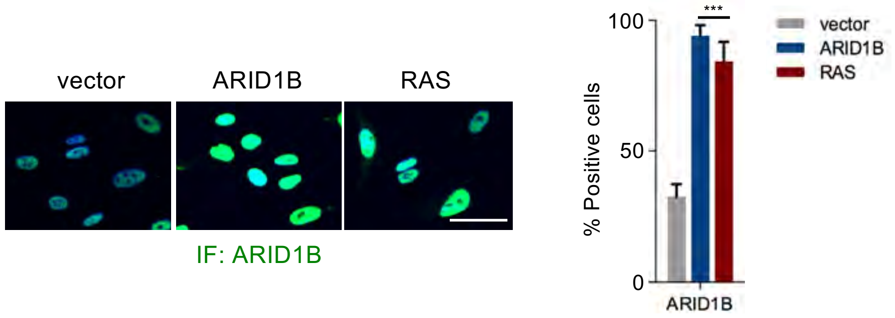

B

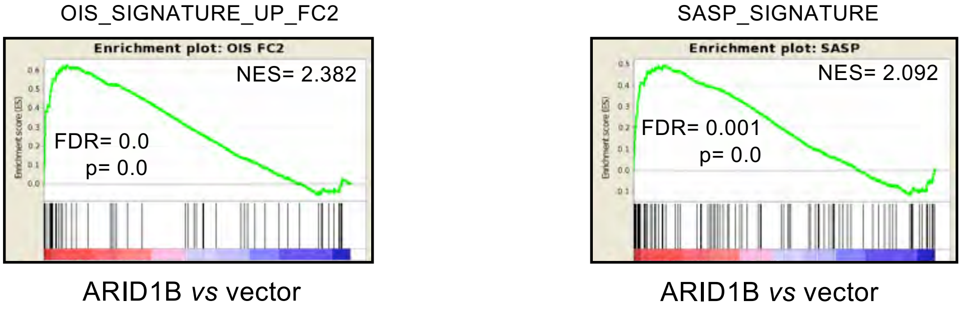

NAME	SIZE	ES	NES	NOM p-val	FDR q-val
EGUCHI_CELL_CYCLE_RB1_TARGETS	22	0.55795574	1.7563792	0.00193424	0.02927713

C

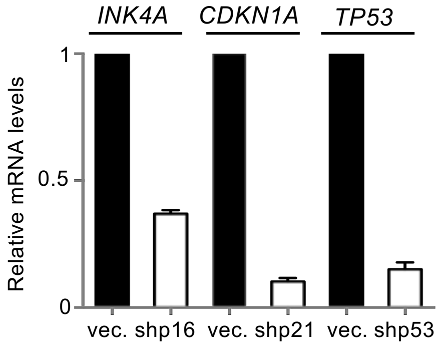

Fig. S5

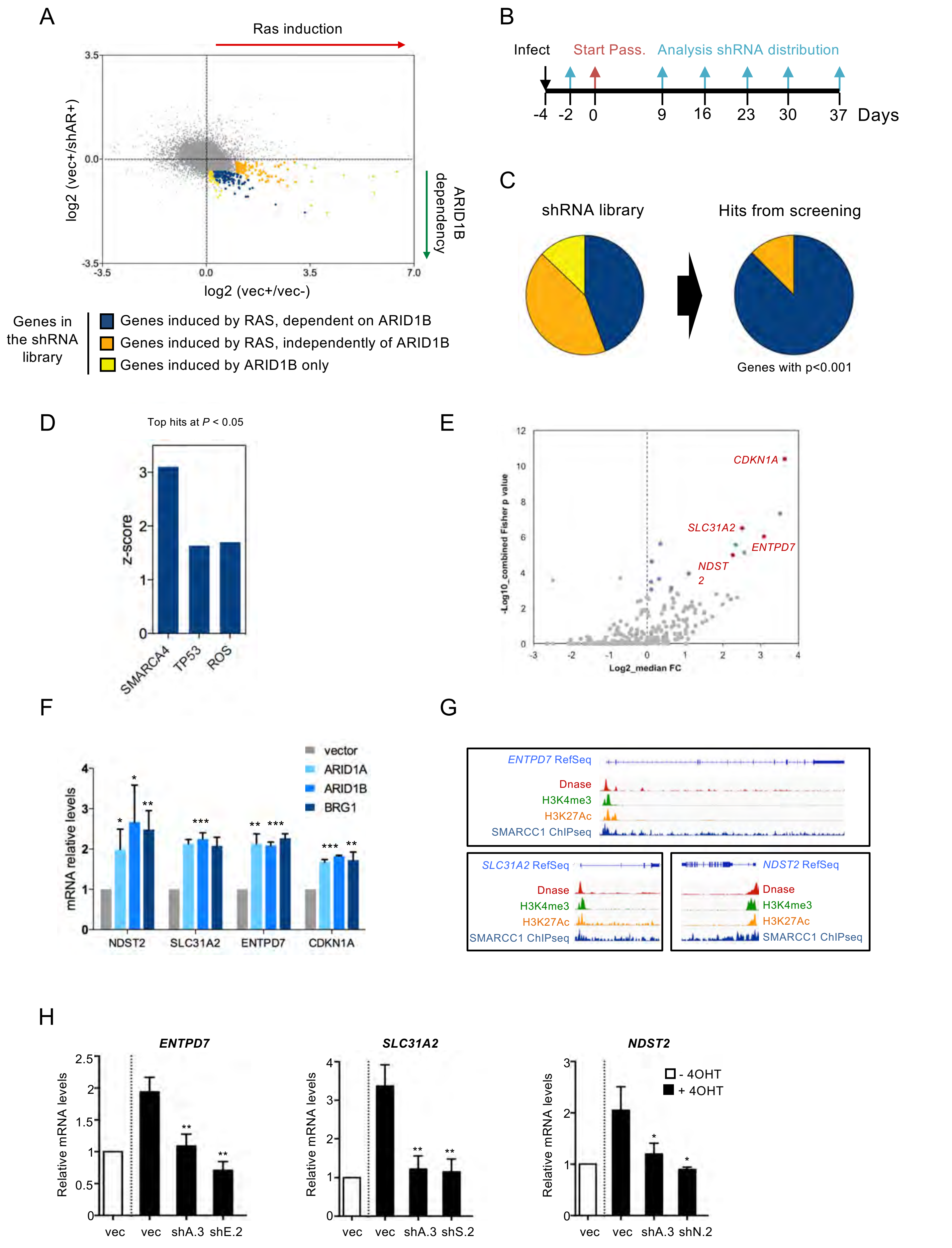

A
Ras induction
log2 (vec+/shAR+)
log2 (vec+/vec-)
ARID1B dependency
Genes in the shRNA library
Genes induced by RAS, dependent on ARID1B
Genes induced by RAS, independently of ARID1B
Genes induced by ARID1B only
B
Infect
Start Pass.
Analysis shRNA distribution
-4 -2 0 9 16 23 30 37 Days
C
shRNA library
Hits from screening
Genes with p<0.001
D
Top hits at P < 0.05
z-score
SMARCA4
TP53
ROS
E
CDKN1A
SLC31A2
ENTPD7
NDST2
-Log10_combined Fisher p value
Log2_median FC
F
vector
ARID1A
ARID1B
BRG1
mRNA relative levels
NDST2
SLC31A2
ENTPD7
CDKN1A
G
ENTPD7 RefSeq
SLC31A2 RefSeq
NDST2 RefSeq
Dnase
H3K4me3
H3K27Ac
SMARCC1 ChIPseq
H
ENTPD7
SLC31A2
NDST2
Relative mRNA levels
- 4OHT
+ 4OHT
vec vec shA.3 shE.2
vec vec shA.3 shS.2
vec vec shA.3 shN.2

Fig. S6

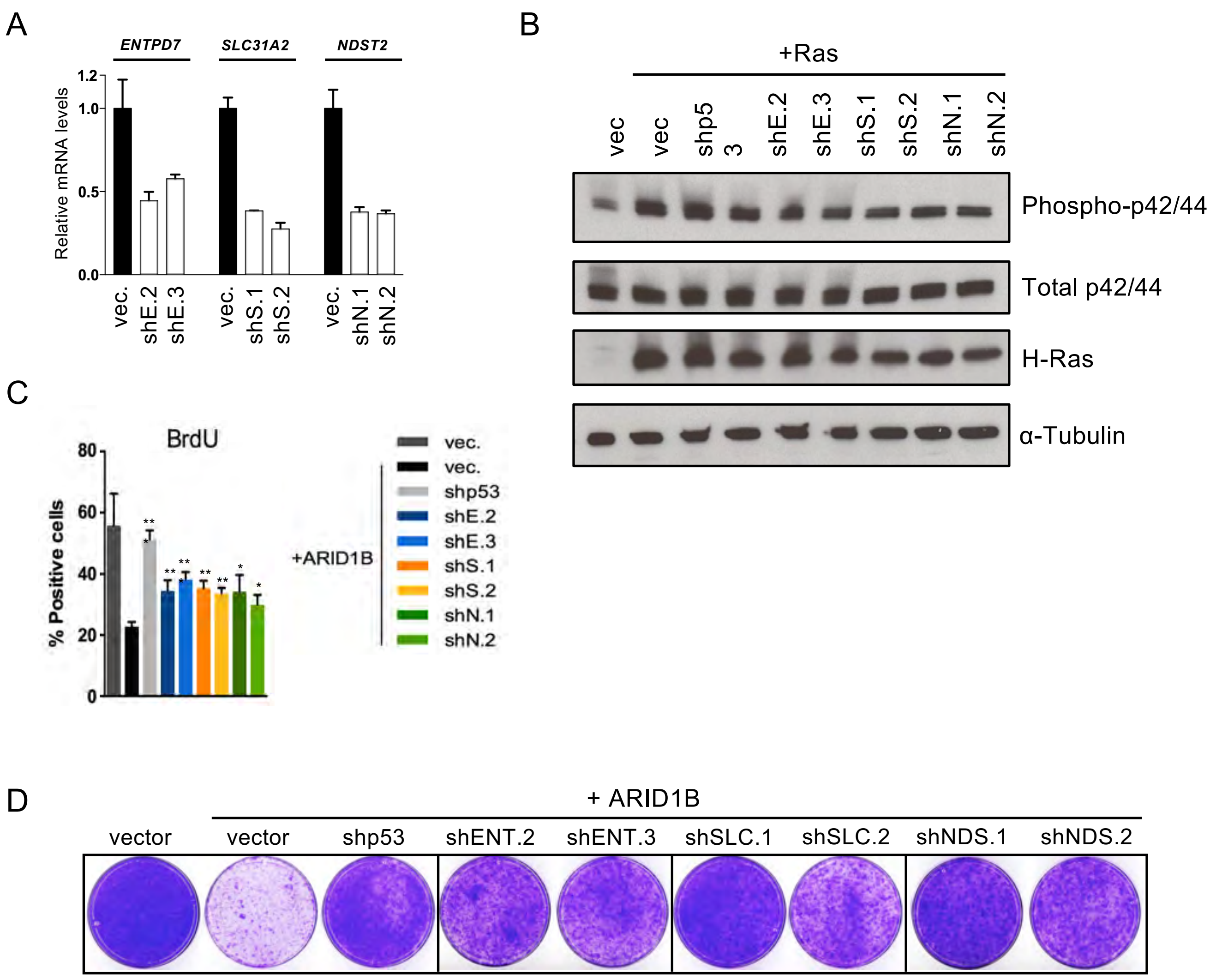
A
ENTPD7
SLC31A2
NDST2
Relative mRNA levels
1.2
1.0
0.5
0.0
vec.
shE.2
shE.3
vec.
shS.1
shS.2
vec.
shN.1
shN.2
B
+Ras
vec
vec
shp5
3
shE.2
shE.3
shS.1
shS.2
shN.1
shN.2
Phospho-p42/44
Total p42/44
H-Ras
α-Tubulin
C
BrdU
% Positive cells
80
60
40
20
0
+ARID1B
vec.
vec.
shp53
shE.2
shE.3
shS.1
shS.2
shN.1
shN.2
D
+ ARID1B
vector
vector
shp53
shENT.2
shENT.3
shSLC.1
shSLC.2
shNDS.1
shNDS.2

Fig. S7

A

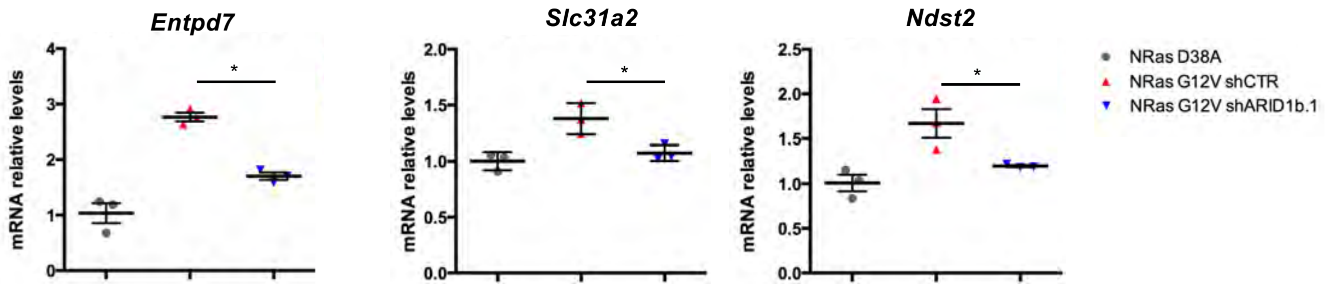

B

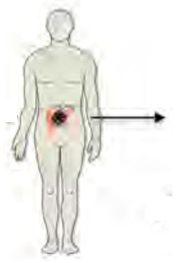

Expression in TCGA LIVER

	Fold Change	P-Val	Loss Gene Rank
ARID1B	-1.106	1.46E-6	Top 11%
ENTPD7	-1.074	1.58E-5	Top 16%
SLC31A2	-1.070	1.14E-5	Top 15%

Normal (115) vs Hepatocellular Carcinoma (97)

C

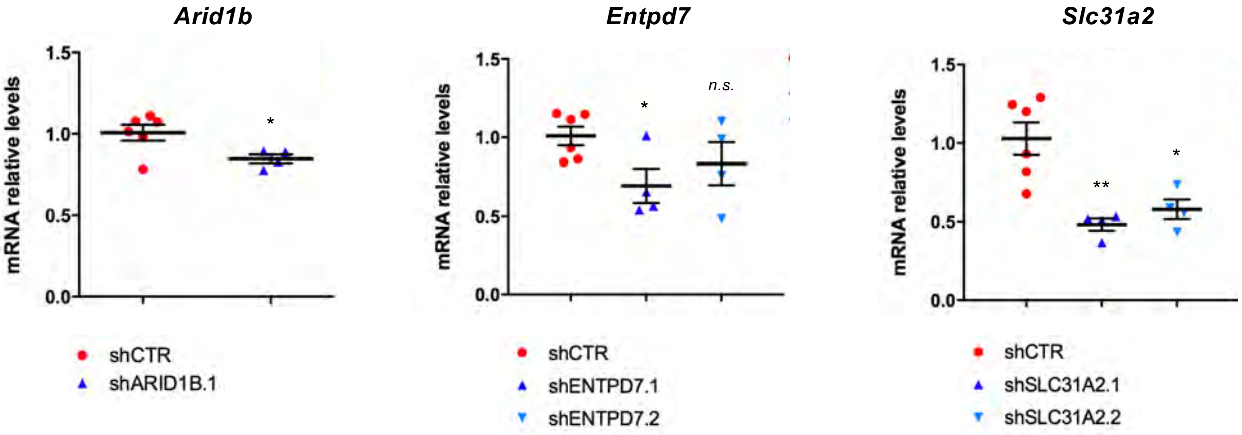

D

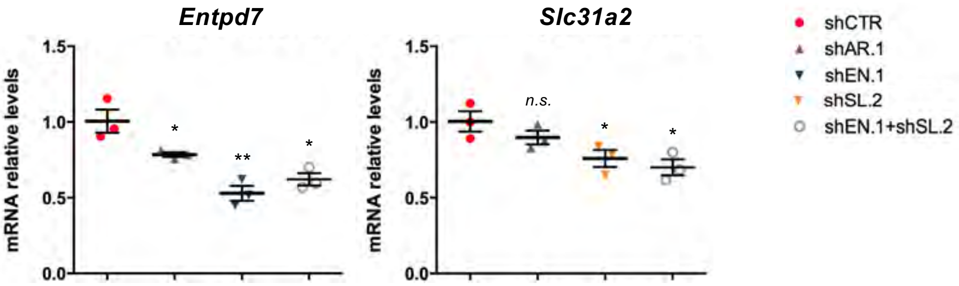

Fig. S8

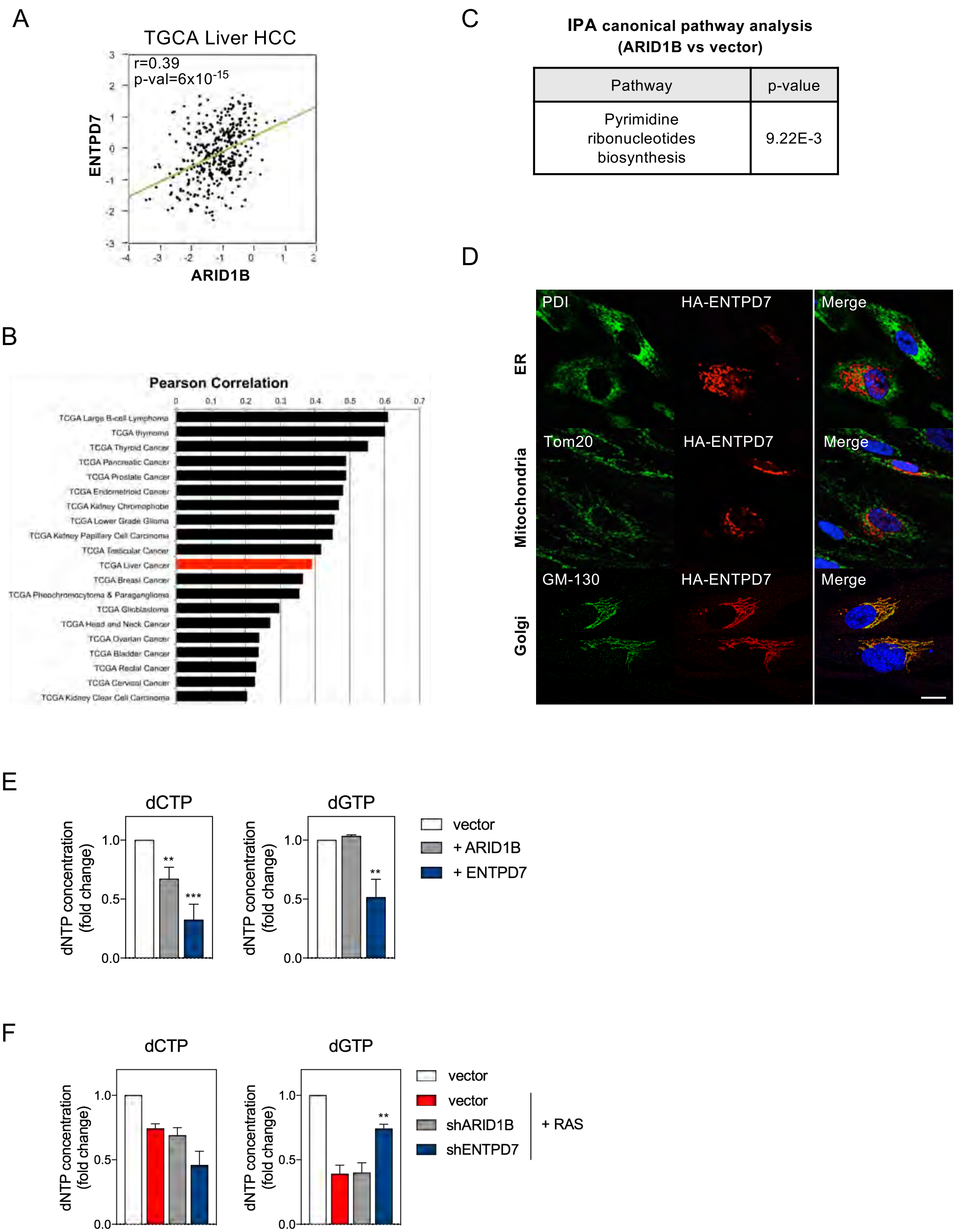

Pathway	p-value
Pyrimidine ribonucleotides biosynthesis	9.22E-3

Fig. S9

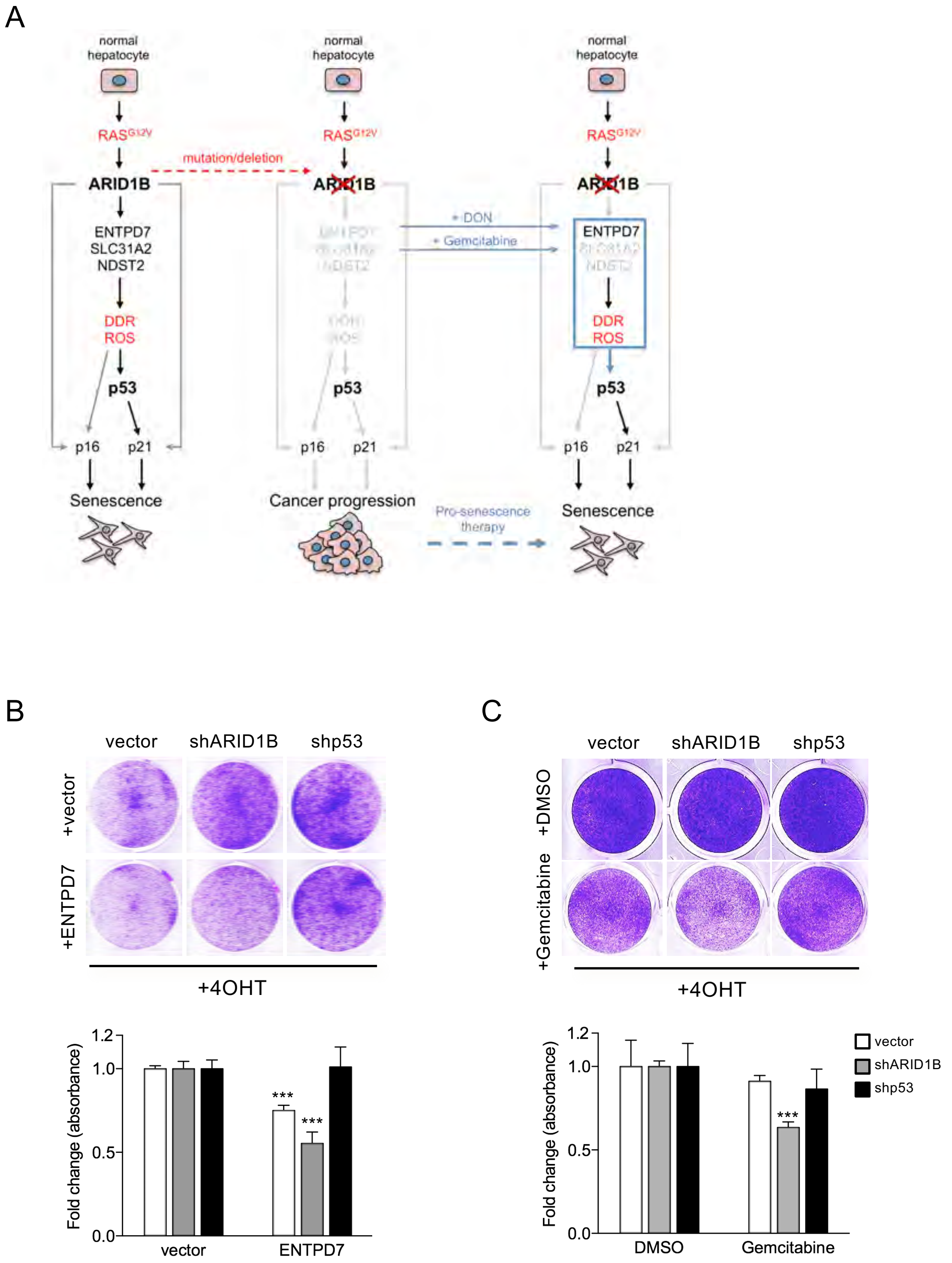

A
normal hepatocyte
RAS G12V
ARID1B
mutation/deletion
ENTPD7
SLC31A2
NDST2
DDR
ROS
p53
p16
p21
Senescence
Cancer progression
+ DON
+ Gemcitabine
Pro-senescence therapy
B
vector
shARID1B
shp53
+vector
+ENTPD7
+4OHT
Fold change (absorbance)
1.2
1.0
0.5
0.0
***
ENTPD7
C
+DMSO
+Gemcitabine
DMSO
Gemcitabine
